# Forgoing earned incentives to signal pure motives

**DOI:** 10.1073/pnas.2000065117

**Published:** 2020-07-06

**Authors:** Erika L. Kirgios, Edward H. Chang, Emma E. Levine, Katherine L. Milkman, Judd B. Kessler

**Affiliations:** ^a^Department of Operations, Information and Decisions, The Wharton School, University of Pennsylvania, Philadelphia, PA 19104;; ^b^Department of Behavioral Science, Booth School of Business, University of Chicago, Chicago, IL 60637;; ^c^Department of Business Economics and Public Policy, The Wharton School, University of Pennsylvania, Philadelphia, PA 19104

**Keywords:** incentives, motivation laundering, self-signaling

## Abstract

Policy makers, employers, and insurers often provide financial incentives to encourage citizens, employees, and customers to take actions that are good for them or for society (e.g., energy conservation, healthy living, safe driving). Although financial incentives are often effective at inducing good behavior, they’ve been shown to have self-image costs: Those who receive incentives view their actions less positively due to the perceived incompatibility between financial incentives and intrinsic motives. We test an intervention that allows organizations and individuals to resolve this tension: We use financial rewards to kick-start good behavior and then offer individuals the opportunity to give up some or all of their earned financial rewards in order to boost their self-image. Two preregistered studies—an incentivized online experiment (*n* = 763) on prosocial behavior and a large field experiment (*n* = 17,968) on exercise—provide evidence that emphasizing the intrinsic rewards of a past action leads individuals to forgo or donate earned financial rewards. Our intervention allows individuals to retroactively signal that they acted for the right reason, which we call “motivation laundering.” We discuss the implications of motivation laundering for the design of incentive systems and behavioral change.

## Significance

Financial incentives can spark behavior change but often damage recipients’ self-image. We designed and tested an intervention that allows organizations and individuals to resolve this tension. We motivated actors with financial rewards and then gave them the opportunity to forgo those rewards to signal their past actions were intrinsically motivated. We propose that actors who forgo financial rewards engage in “motivation laundering,” passing up payments earned for an incentivized action to retroactively signal that their motivations were intrinsic. Our intervention has the potential to leave organizations and incentivized individuals better off: Financial rewards help actors build better habits, and motivation laundering allows them to boost their self-image, while giving organizations opportunities to lower incentive program costs.

Policy makers, employers, and insurers frequently reward people for behavior that is good for them or for society. The government provides tax deductions for charitable giving; car insurance companies offer up to 35% discounts for college students who maintain at least a B average (1); in most of the country, smokers pay roughly 350% more for life insurance than nonsmokers and are subject to health insurance surcharges of up to 50% (2, 3); utility companies offer rebates to customers who install energy-efficient heating and cooling systems (4); and 72% of large organizations offer incentive-based wellness programs that encourage employees to get health screenings and visit the gym (5). In the absence of financial incentives, these behaviors—donating to charity, getting good grades, abstaining from cigarettes, conserving energy, visiting the doctor, and exercising—might make people feel proud, allowing them to signal to themselves and others that they genuinely care about bettering themselves and the world.

But do individuals feel as good about these actions if they have been paid to take them? Past research suggests that when an actor accepts a financial reward for taking a positive action, observers view the action and the actor’s motives for taking it with skepticism (6, 7). People believe material and reputational rewards fundamentally conflict with intrinsic motives (8–10). As a result, financially motivated actions are perceived as less authentic and less diagnostic of an actor’s true character than intrinsically motivated actions (7, 11).

When choosing how to behave, people seem to anticipate the perverse effects of incentives. In public, they exert less effort on prosocial behavior when the behavior is incentivized than when it is not (9, 12–15). In private, they decrease effort on intrinsically rewarding activities (e.g., puzzle solving, studying) when they are incentivized (16–19). These results suggest that financial incentives undermine actors’ ability to signal—to others or to themselves—that their good behaviors imply they are good people.

While others have explored how incentives affect effort in the presence of signaling concerns, we posit that, even after effort has been exerted, perceptions of actors’ motives for exerting that effort may remain malleable. If true, those paid to engage in “good” behavior (i.e., behavior that is seen as positive, socially desirable, and intrinsically rewarding) may want to disavow the financial incentives previously earned for their good behavior, particularly when the intrinsic value of the behavior is highlighted. By doing so, they can create a more favorable interpretation of why they engaged in the behavior in the first place. Put differently, we hypothesize that actors who were promised rewards for good behavior ex ante may choose to forgo those rewards ex post so they can retrospectively interpret their good behavior as stemming from intrinsic motives.

This hypothesis is consistent with past research suggesting that people value opportunities to engage in psychological money laundering or opportunities to “cleanse” money earned through unethical behavior by giving part of it up to a good cause or by pooling it with money earned through ethical means (20–22). People seek to recategorize their “dirty” money as ethical money to repair some of the self-image damage from their original unethical behavior (23). We propose that people not only engage in psychological money laundering for unethical behavior, but they also engage in “motivation laundering” for incentivized good behavior, attempting to recategorize their motives as intrinsic ex post. We suggest that the opportunity to motivation launder will be more salient and appealing when the intrinsic rewards of the virtuous behavior are emphasized, leading more participants to give up their incentives to launder their motives.

Of course, giving up financial incentives comes with a monetary cost. With this in mind, we hypothesize that reminding people of the intrinsic rewards accrued from their past actions will increase their willingness to sacrifice financial incentives to signal intrinsic motives more under some conditions than others. First, when the intrinsic motives in question are more consistent with the values actors hold and the self-image they hope to project, we expect such reminders to be particularly potent (24–26). Second, we expect that the effect of emphasizing intrinsic rewards will be stronger for actors who have expended significant effort on the incentivized good behavior. This is because the signaling value of forgoing incentives should be larger for participants who exerted more effort given that costly and effortful actions are seen as more diagnostic of personal traits and preferences (25, 27, 28). Third, the sooner the opportunity to forgo incentives arrives after an incentivized action is completed, the more appealing we expect this opportunity to be because the signaling process can be muddied over time by forgetfulness or intervening events (26, 29, 30). In all of these cases, actors have more to gain from signaling that their motives were intrinsic and more to lose if their motives are seen as tainted by financial incentives.

In this paper, we present results from two preregistered experiments showing that prompting people to recognize the intrinsic benefits of incentivized good behavior makes them more willing to forgo incentives earned for that behavior. Our prompts highlight an opportunity for people to self-signal that they completed a virtuous act for the “right” reasons and that their motives were intrinsic rather than financial. The prompts may lead people to reject cash rewards by encouraging those who behaved virtuously for the “right” reasons to reaffirm this was the case. Or, they may operate on guilt, leading people who acted virtuously for cash rewards to feel as if they had the “wrong” motives. Past research suggests guilt can motivate self-signaling (31, 32). However, if people returned incentives in response to our prompts to alleviate guilt, it might suggest our prompts left them worse off, overall. We theorize and offer evidence suggesting that this is unlikely to be the case. Instead, the opportunity to motivation launder by forgoing incentives (highlighted by our prompts) allows people to earn a self-image boost that outweighs any costs of guilt invoked by our treatment.

While it is important to note that social signaling may also influence decisions about forgoing incentives, our primary theoretical focus here is on self-signaling because the decision-making we study occurs in private. However, individuals may want to signal both to themselves and to others (e.g., the experimenter) that their motives were intrinsic.

Our first study involved 763 online participants who were paid $2.00 to write a hopeful, kind letter to a hospitalized child over the holidays. After writing the letter, participants were randomly assigned to one of three experimental conditions. In the first condition, we encouraged participants to treat the intrinsically rewarding features of their experience (the joy and hope they spread) as their reward. In the control condition, we gave them no additional prompting. In a second control condition, designed to test our hypothesis that people are particularly likely to forgo financial rewards when reminded of intrinsic rewards that are consistent with the image they want to project to themselves and others, we prompted participants to treat a less desirable benefit (practice with letter writing) as their reward.* We then gave participants the option to keep or forgo their earnings. Participants who were reminded of the desirable intrinsic rewards from the letter-writing activity gave up more money than those in either of our control conditions. Consistent with our hypotheses, this effect was moderated by the time participants spent on the letter-writing task, our proxy for the effort participants exerted on the task. Further, we found that participants were just as willing to repeat the experimental letter-writing task in our treatment condition as in our control conditions. Thus, if our treatment induced guilt, it was not meaningful enough to decrease willingness to repeat the same experience.

In a second study extending this finding to a policy-relevant field setting, we ran a preregistered field experiment with members of a national gym chain (*n* = 17,968). All participants in this field experiment had just completed a 4-wk program designed by a nonprofit to promote exercise and had earned monetary incentives for participating. After completing the program, participants were given the choice between keeping their earnings or forgoing their earnings (by donating them back to the nonprofit). Participants were randomly assigned to either a treatment or control condition. In the treatment condition, participants were encouraged to treat the intrinsic, nonmonetary rewards they had earned from the program (“the healthy habits you’ve kick-started”) as their reward and donate their earnings. In the control condition, participants were simply encouraged to donate their earnings without any such reminder of the intrinsic rewards they had earned. Participants were significantly more likely to forgo their earnings in the treatment condition, though the effect size was modest: The treatment condition increased donation rates by 1.04 percentage points relative to the control condition. As predicted in our preregistration, the effect of emphasizing intrinsic rewards was strongest among participants who had exercised most frequently during the rewards program. This finding is consistent with our self-signaling account: People who exercised the most had the most to gain by signaling intrinsic motives. These high-frequency exercisers were most responsive to our treatment despite the fact that they had earned more during the exercise program’s incentive period and therefore had to incur a larger (financial) cost in order to signal pure motives. Finally, consistent with our hypotheses, we found that our treatment effect decayed with time: Longer time delays between program completion and an invitation to forgo earnings were associated with a reduction in our treatment effect.

These studies provide evidence that people willingly forgo financial incentives earned for good behavior and are more eager to do so when reminded of the nonmonetary, intrinsic benefits of the behavior. Study 1 focuses on a moral domain (writing letters to sick children), highlights an intrinsic reward that benefits others (spreading joy and hope), and asks participants to forgo their earnings without specifying a recipient for the forgone cash. Study 2, a field experiment, focuses on a self-improvement behavior (exercising), emphasizes an intrinsic reward that benefits the self (building healthy habits), and asks participants to donate their earnings to a specific recipient (the nonprofit that rewarded them in the first place). Taken together, these studies demonstrate that our effect holds both in the laboratory and in the field, for both prosocial and self-improvement behaviors, and for both intrinsic rewards that come from helping others and from helping oneself. Furthermore, our studies suggest that people will engage in motivation laundering both by donating their earnings and by simply giving them up.

Our work also highlights dual potential benefits from providing individuals with an opportunity to forgo financial incentives offered for past actions. Financial incentives have been robustly shown to motivate good behaviors, even in domains in which intrinsic motives are high (33–35). However, those who have or value intrinsic motives—particularly those who see those motives as part of their identity or as reflecting a positive self-image—might prefer to return the incentives when those intrinsic motives are highlighted. That is, offering individuals rewards to spur their motivation and subsequently offering them the chance to return those rewards may allow them to bolster their self-image. Our data suggest that our treatment does not have adverse effects on individual well-being, and we speculate that giving participants the opportunity to boost their self-image by forgoing their incentives may even leave them better off. Furthermore, allowing individuals to give up their promised incentives after they have acted—and using these returned incentives to motivate others—can help organizations motivate good behavior more efficiently.

## Study 1: Online Experiment

### Method.

Seven hundred seventy-five participants were recruited through Prolific to participate in a preregistered (see https://aspredicted.org/y5vt8.pdf) study in exchange for $0.50.^†^ Study participants were first asked to complete a short image classification task irrelevant to the remainder of the study. Then they were given the choice to opt in to complete a second task for a $2.00 bonus. The 763 participants who opted to complete the second task (46.7% female) were randomly assigned to a condition and included in our analysis, following our preregistration.^‡^ All studies included in this paper were approved by the University of Pennsylvania Institutional Review Board, and all subjects gave informed consent.

All 763 participants were asked to write a letter to a sick child who would be spending the upcoming holiday season in the hospital. We explained that the goal of each letter was to provide hope to a sick child. Participants were reminded that they were being paid $2.00 to write these letters and then were instructed to write a letter. We recorded the time each participant spent crafting their letter as a proxy for effort. All letters deemed appropriate will be sent to sick children via the I See Me! Letters of Love campaign in December 2020, in partnership with the Children’s Cancer Research Fund.

After participants wrote their letters, they were randomized into one of three experimental conditions: a treatment condition, an active control condition, or a baseline control condition. In all conditions, participants were given the opportunity to forgo some or all of their $2.00 earnings from writing the letters.^§^ In the treatment condition, we reminded participants of the intrinsic rewards associated with the letter-writing activity before suggesting that they forgo their monetary rewards. Specifically, we prompted them to “treat the joy and hope you’ve spread as your reward” before inviting them to forgo some or all of their bonus. In the active control condition, participants were reminded of an alternative, less-valued reward of the letter-writing activity, which we did not expect participants to see as a meaningful motivator. Specifically, we prompted them to “treat the letter-writing practice you’ve received as your reward” before inviting them to forgo some or all of their bonus. This active control condition was meant to control for possible experimenter demand effects by asking participants to forgo payment for a benefit that was unlikely to be seen as a central or valued motivator of the letter-writing activity. It was also meant to test our hypothesis that value-congruent intrinsic benefits would be more likely to increase the rate at which participants gave up their incentives.^¶^ In the baseline control condition, participants did not receive any additional prompts before they were invited to forgo some or all of their bonus.

To ensure that our treatment did not make participants worse off (e.g., by inducing guilt), we next measured participants’ willingness to repeat the experimental experience they had just completed. After participants decided how much of their bonus to forgo, we asked participants: “Would you be willing to participate in more studies like this one for $1?” Participants who responded affirmatively to this question were offered the opportunity to repeat the study experience 1 mo later.

Finally, participants responded to two exploratory questions meant to measure their motivation for writing letters. They indicated their agreement on a scale from “1 (Not at all)” to “7 (Extremely)” with the following two items: 1) “I engaged in the letter-writing task primarily to help a child in need” and 2) “My decision to write a letter to a child in need was authentic” [see *SI Appendix* for more details on exploratory measures and analyses and to view all study materials from study 1; all data and code from study 1 are available on OSF (36)].

### Results.

**Fig. 1.**
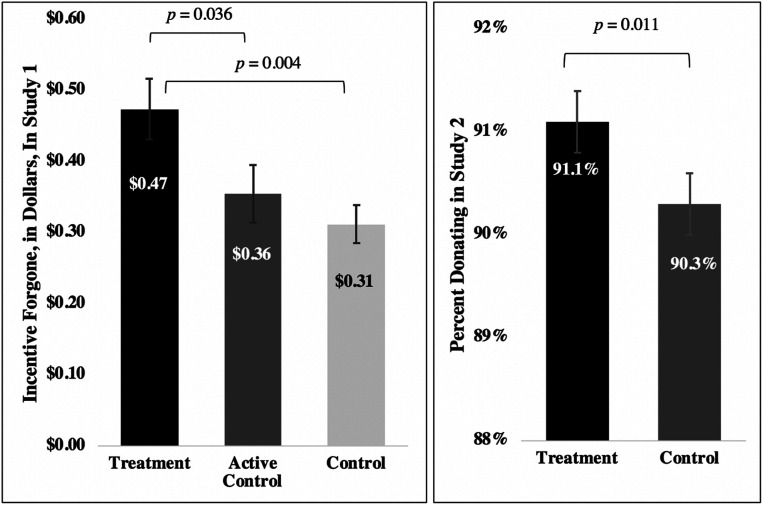
This figure depicts the effects of our treatment on willingness to forgo incentives in studies 1 and 2. The *Left* plots the mean dollars forgone by participants in each of the three conditions in study 1 (participants could forgo between $0 and $2). SE bars are depicted around each mean. The *Right* shows the proportion of study 2 participants donating their incentive payment in the treatment and control conditions, respectively (in study 2, participants were faced with a binary decision about donating). Participants who did not reply to an email in study 2 inviting them to opt out of donating automatically became donors, which is why donation base rates are so high. SE bars are depicted around each mean.

Our primary dependent variable of interest was the quantity of a participant’s forgone earnings (a value that could range from 0 to 200 cents). Participants gave up 47.4 cents on average (SD = 68.3) in the treatment condition, 35.6 cents on average (SD = 63.6) in the active control condition, and 31.2 cents on average (SD = 59.4) in the baseline control condition (Fig. 1). When examining whether participants gave up any earnings at all, we see that 40.6% gave up some or all of their earnings in the treatment condition, while 29.0% and 27.1% chose to forgo some or all of their earnings in the active control condition and the baseline control condition, respectively.

Following our preregistered analysis plan, we ran an ordinary least squares regression to predict each participant’s forgone earnings. The only predictor variables in this regression were indicators for experimental conditions, and the indicator for our baseline control condition was omitted. Assignment to our treatment condition significantly increased forgone earnings by $0.16 (or 51.9%, *P* = 0.0043) relative to assignment to the baseline control condition. A Wald test confirmed that assignment to the treatment condition also significantly increased forgone earnings by $0.12 (or 33.4%, *P* = 0.036) relative to assignment to the active control condition, and the difference between being in the active and baseline control conditions was not significant (*P* = 0.405). See *SI Appendix*, Table S1 for complete regression results.

These results support our prediction that people will forgo monetary incentives they have already earned when reminded of the intrinsic, nonmonetary rewards of their actions. Furthermore, our effect does not appear to arise due to an experimenter demand effect, given that reminding people of intrinsic rewards that were not value-congruent did not change their behavior. These results further suggest that participants’ willingness to give up their earnings is driven by self-image concerns as the rate of sacrificing earnings only increased when we highlighted motives that would promote a positive self-image. In a supplementary study, we found further support for the hypothesis that value-congruence moderates our treatment effect: Participants who self-reported being more motivated by the prosocial rewards of the letter-writing task showed a larger treatment effect (*SI Appendix, Study S1* and Fig. S5).

Next, we tested whether our treatment reduced participants’ willingness to complete a similar study in the future. If participants were less willing to repeat the experience in the treatment condition than in the control conditions, it suggests our treatment may have produced negative side effects, such as guilt. As preregistered, we ran a regression with robust SEs where willingness to opt in to future letter-writing tasks like the one participants had just completed was the dependent variable, and the predictor variables were separate indicators for being in the treatment and active control conditions.

We found no significant differences in willingness to repeat the task by condition: 91.8% were willing to repeat their experience in the treatment condition, 92.5% in the active control condition, and 88.2% in the baseline control condition. Wald tests demonstrated that there was no difference in willingness to repeat the experience at standard significance levels between the treatment and the active control condition (*P =* 0.796), the active control condition and the baseline control condition (*P* = 0.100), or the treatment and the baseline control condition (*P* = 0.163). Notably, participants in the treatment condition were directionally more willing to repeat the experience than those in the baseline control condition, though this effect was not significant. These results suggest that our treatment did not produce adverse consequences for participants even though participants in the treatment condition earned less in the study. Indeed, the data suggest participants in the treatment condition were just as willing to repeat their experimental experience for less money (because they gave up more money on average), suggesting that the self-image boost they gained made up for the financial cost of forgoing their incentives.

We also examined whether participants who exerted more effort on the letter-writing task (measured by time spent on the task) were more responsive to our treatment. This analysis was exploratory and was not preregistered. We ran an ordinary least squares regression predicting incentives forgone with the following predictor variables: indicators for experimental condition (baseline control indicator omitted), a mean-centered measure of time spent on the letter-writing task, and interactions between each condition indicator and mean-centered time spent writing. Consistent with our hypothesis, the interaction between time spent on the letter and assignment to our treatment condition was significant and positive (*b* = 0.125, SE = 0.055, *P* = 0.022). Specifically, we found that a one SD increase in time on the task (or 260 extra seconds spent writing) was associated with a 77.2% increase in the estimated treatment effect. See Table 1 for complete regression results, and see *SI Appendix, Robustness of the Interaction between Time Spent Composing a Letter and Assignment to Our Treatment Condition* for robustness checks.

**Table 1.** Regression-estimated effects of our treatment on forgone incentives in study 1

	How much of the incentive was returned? (in dollars)	
	Model 1	Model 2
Active control	0.043 (0.057)	0.045 (0.056)
Treatment	0.162** (0.057)	0.173** (0.056)
Letter-writing time		0.022 (0.034)
Active control* Letter-writing time		0.012 (0.058)
Treatment* Letter-writing time		0.125* (0.055)
Observations	763	763
Adjusted *R*^2^	0.009	0.081

This table reports the results of two ordinary least squares regressions predicting how much money, in dollars, participants in Study 1 chose to forgo (from $0 to $2), with indicators for whether they were in the treatment condition or the active control condition. Model 2 includes a mean-centered measure of the time participants spent writing a letter to a sick child as an independent variable, as well as interactions between this measure and indicators for our treatment condition and active control condition. Robust SEs are in parentheses. * and ** denote significance at the 5% and 1% levels, respectively.

## Study 2: Field Experiment

In study 2, we tested whether emphasizing the intrinsic rewards of completed incentivized actions would also increase the rate at which individuals gave up their incentives in a policy-relevant field context. We also tested whether this effect would hold for a self-improvement behavior rather than a prosocial behavior.

### Method.

We conducted a preregistered (see https://aspredicted.org/i6xy5.pdf) field experiment in partnership with a program called StepUp, a 28-d digital rewards program designed to promote exercise among members of 24 hour Fitness, a large, American gym chain. All StepUp program participants earned monetary incentives during the program (average earnings were $2.04; 95% earned between $0.08 and $5.65). After completing the StepUp Program, 17,968 people^#^ (31.5% of whom identified as male, 1.6% of whom did not identify their sex) were selected for inclusion in our experiment. Eligibility for our experiment was based on registering for the StepUp program between 3 April 2018 and 16 August 2018.

Participants in our study were randomly assigned to either a treatment or control condition. Everyone received an email inviting them to either claim their StepUp earnings or donate those earnings back to the StepUp program, but this call for donations included an additional prompt in the treatment condition. Specifically, participants were prompted to “treat the healthy habits you’ve kick-started as your reward” by donating their earnings back to the StepUp program (see *SI Appendix* for exact study stimuli). Study emails were sent to participants in batches, and participants received them an average of 33.79 d after completing the StepUp program (minimum wait = 3 d; maximum wait = 70 d; SD = 12.61 d; see *SI Appendix*, Fig. S2 for the distribution of wait times; all study materials from study 2 are available in *SI Appendix*, and all data and code from study 2 are available on OSF (36).

### Results.

Our dependent variable of interest was whether participants kept their earnings. A total of 91.14% of participants donated their earnings in the treatment condition compared to 90.27% in the control condition (Fig. 1).^||^ Following our preregistered analysis plan, we ran an ordinary least squares regression with robust SEs to predict a participant’s decision to donate their earnings. This (preregistered) regression included a control for the amount earned by the participant in the StepUp program, fixed effects for the version of the StepUp program the participant had been randomly assigned to experience, an indicator for being male, and an indicator for “unknown sex.” (For those participants who had missing data for sex, a research assistant [RA] attempted to label their sex based on their name. The RA used an algorithm that pairs names with their most likely sex and used Google searches when this algorithm did not work. The 1.6% of individuals with unknown sex are those with names that could not be identified by the RA.) As predicted, we found that participants were significantly more likely to donate their earnings in the treatment condition than in the control condition (estimated increase in likelihood of donating earnings: 1.04 percentage points, 95% CI [0.24, 1.84]; *P* = 0.011; see Table 2, Model 1 for complete regression results). This result is robust to the removal of our preregistered covariates (see *SI Appendix*, Table S2, Models 1 to 3). While this treatment effect is objectively small, our test is fairly conservative: To the extent that open rates are low, the “treatment on treated” effect may be fairly substantial (e.g., if only 20% of recipients open the email, the effects on those who open it are five times as large).

**Table 2.** Regression-estimated effects of our treatment on donated incentives in study 2

	Was the incentive donated? (1 = yes, 0 = no)	
	Model 1	Model 2
Treatment	0.010* (0.004)	0.011** (0.004)
Cash earned	−0.029*** (0.001)	−0.017*** (0.001)
Male	0.009* (0.004)	0.020*** (0.004)
Sex unknown	−0.011 (0.017)	−0.010 (0.016)
Visits (during incentive period)		−0.059*** (0.004)
Treatment* Visits		0.010* (0.005)
StepUp program fixed effects	Yes	Yes
Observations	17,968	17,968
Adjusted *R*^2^	0.111	0.131

This table reports the results of two ordinary least squares regression models predicting whether a StepUp participant in our experiment chose to donate their incentives in study 2 (1 = yes, donated; 0 = no, did not donate). Model 1 shows the main effect of our treatment while model 2 shows moderation. Predictors in model 1 include an indicator for being assigned to the treatment condition, fixed effects for a participant’s StepUp program, the amount of cash earned by the participant, and indicators for the participant’s sex (female omitted). Model 2 includes two additional predictors to test for moderation. These additional predictors are the (mean-centered) number of times the participant visited the gym during the StepUp program and an interaction between this variable and assignment to our treatment condition. Robust SEs are in parentheses. *, **, and *** denote significance at the 5%, 1%, and 0.1% levels, respectively.

We also examined whether the treatment effect was stronger for participants who had exercised more during the StepUp program, as hypothesized. Following our preregistration, we measured exercise by tallying the number of times each participant visited the gym during the StepUp program. We ran the same ordinary least squares regression described above but added mean-centered total workouts during the StepUp program as a main effect, as well as an interaction between this variable and our treatment indicator.** We found that for every extra gym visit a participant made during the exercise program, the impact of our treatment on the decision to donate increased by 99%, or roughly 1.03 percentage points, and this interaction was marginally significant (*P* = 0.049; see Table 2, Model 2 for complete regression results and *SI Appendix*, Figs. S3 and S4 to visualize the interaction between exercise frequency and the treatment condition).^††^ This result is robust to removal of our preregistered covariates (*SI Appendix*, Table S3, Models 1 to 3)^‡‡^ and is consistent with our prediction, though it is also consistent with the possibility that more frequent gym goers generally value fitness more and are thus more motivated to donate their earnings to help others reap intrinsic rewards from exercise.^§§^

Our analyses also show a negative overall relationship between exercise frequency and giving. This is unsurprising because frequent gym visitors earned more money in StepUp, and because expending effort can increase the subjective value of earned incentives (37). Thus, we would expect frequent gym visitors to be less willing overall to give up their earnings across experimental conditions—as they earned higher value incentives—even though they are more sensitive to our treatment. Indeed, our findings show that the negative relationship between gym visit frequency and giving is greatly reduced when controlling for incentive earnings, and the fact that the relationship remains negative is consistent with effort increasing the subjective value of earned incentives. It is also possible that other differences between high and low frequency exercisers may explain the negative relationship between exercise frequency and donations in study 2.

Finally, because the signaling value of a forgone incentive may decline over time (29, 30), in an exploratory analysis, we exploited random variation in the time lag between StepUp program completion and when participants received our email to test the hypothesis that our treatment effect would decay as the time lag increased. We ran an ordinary least squares regression with robust SEs to predict whether participants donated their earnings. Predictors in our regression included an indicator for being in the treatment condition, a continuous variable representing the time delay (in days) between program completion and email receipt, and an interaction between the two. As in our preregistered analyses, we included a control for the amount earned by the participant in the StepUp program, fixed effects for the version of the StepUp program the participant had been randomly assigned to experience, an indicator for being male, and an indicator for “unknown sex.” As predicted, each extra week that passed between program completion and email receipt decreased the size of our treatment effect by 47%, or 0.49 percentage points (*P* = 0.026; see *SI Appendix*, Table S4 for complete regression results).^¶¶^

## Discussion

Although financial incentives for good behavior are common, people tend to judge good behaviors less positively in themselves and in others when they believe those behaviors were motivated by monetary rewards (7, 8, 10, 38). We provide evidence that this tension between extrinsic and intrinsic rewards can lead people to forgo already earned financial incentives for good behavior when reminded of that behavior’s intrinsic value. We show this in an incentivized, preregistered online experiment and a large-scale, preregistered field experiment. These experiments demonstrate that the effect of emphasizing intrinsic rewards on willingness to forgo earnings is robust and generalizable. Our effect holds for both prosocial and self-improvement behaviors. We show that actors are willing to forgo some or all of their earnings when reminded of intrinsic rewards that benefit others and those that benefit themselves, so long as the reward is value-congruent. We argue that the willingness to forgo earnings we document stems from self-image concerns: People feel better about their past actions when they believe that they were intrinsically motivated, and giving up some or all of their earnings allows them to recast their perceptions of their own motives. Consistent with this hypothesis, we find that the effect of emphasizing intrinsic rewards is stronger when the potential self-image gains of giving up incentives are larger (i.e., when the intrinsic rewards emphasized are value-congruent and when the actor exerted more effort).

Our findings extend past research on psychological money laundering, which has shown that people attempt to “cleanse” money earned in unethical ways by spending some of it on charitable giving or pooling it with money obtained ethically to obfuscate its origin (20–22). While psychological money laundering helps people protect their self-image after receiving ill-gotten gains, our work demonstrates that people may be similarly motivated to “cleanse” their motives for good behavior. Being reminded of the intrinsic rewards associated with an incentivized activity highlights that the good behavior could be cast in a more positive light if the incentives had not been present. We show that actors are then willing to forgo earned incentives for good behavior to “cleanse” themselves of any perceived impure motives.^##^ Thus, our findings extend past research on psychological money laundering, suggesting people engage in motivation laundering as well.

Our work also contributes to the literature on the perceived incompatibility between financial and intrinsic motives (8, 9) by offering evidence that an actor’s perceptions of their motivations are malleable even after they have acted, and that actors will capitalize on this malleability by taking opportunities to retroactively signal their pure motives. In doing so, we extend past research on self-signaling, which has shown that costly actions are taken to signal positive personality traits or preferences in the present moment (24, 39). Prior self-signaling work does not allow for current actions to modify the signal that people receive from their past behavior; rather, prior work finds that people’s signaling decisions modify their perceptions of current behavior and may influence future behaviors due to a desire for consistency (9, 24, 40, 41). We provide evidence that people also engage in retroactive self-signaling, incurring financial costs in the moment to signal new information about their past selves and reconstrue their past actions.

Our findings raise a number of interesting questions for future research. For example, would our treatment be just as effective if participants were forewarned about the opportunity to give up their incentives? And would it work as well if used repeatedly over time? Prior work suggests that being forewarned about the opportunity to donate to charity decreases prosocial behavior (42), suggesting that our treatment effect might wane if participants were forewarned about the opportunity to forgo their earnings. Another open question raised by our findings is whether our treatment increases people’s willingness to complete the incentivized activity again in the absence of monetary rewards. Exploring these questions would teach us more about the durability and equilibrium implications of inviting people to engage in motivation laundering.

The perceived incompatibility between financial incentives and intrinsic motivation (7, 8) creates a tension for actors between 1) earning payments for good deeds (payments that might spur them to take an action that they otherwise would not take) and 2) a desire to feel that they have acted on intrinsic motives. We demonstrate a way to resolve this tension. Our findings suggest a strategy that organizations can use to encourage positive behavior change using incentive programs that still gives actors the opportunity to feel that their motivations were intrinsic. Financial rewards may be the spark that kick-starts good behavior, but giving actors the opportunity to give up earnings ex post may leave them with good behavior and a boost to their self-image while also lowering the cost of incentive programs overall.

### Data Availability.

All study materials are available in *SI Appendix*. Code and deidentified data are available on the Open Science Framework (OSF) (36).

## Supplementary Material

Supplementary File

Supplementary File

Supplementary File

## Footnotes

*We pretested this benefit to confirm that participants saw it as less central to the letter-writing task and less value-congruent than the intrinsic reward mentioned in the treatment condition (the joy and hope they spread). See footnote ¶ for more details.

^†^Before enrolling in the study, Prolific respondents were asked two attention check questions. Those who failed to answer both questions correctly were not allowed to proceed to our study. They were not randomized into an experimental condition and are not included in our count of 775 participants.

^‡^Before being randomized into an experimental condition, these participants were required to correctly answer a second pair of comprehension check questions. They could attempt these questions as many times as necessary before continuing on with our study, and everyone eventually moved forward.

^§^We were intentionally vague about the forgone money. We did not specify a recipient for the money, and we did not imply the money would support more letter writing. The only unambiguous value of giving up the incentive was thus the self-signaling value.

^¶^In a pretest of our stimuli with 100 subjects recruited on Amazon’s Mechanical Turk, we found that participants rated spreading joy and hope as a worthier motivation for the letter-writing behavior (M_joy_treatment_ = 4.48) than gaining practice writing letters (M_practice_control_ = 2.88, *t*(98) = 10.08, *P* < 0.001), and participants stated that they would feel more proud of themselves for writing the letter if they were motivated by spreading joy and hope (M_joy_treatment_ = 4.15) than if they were motivated by letter-writing practice (M_practice_control_ = 2.80, *t*(98) = 7.34, *P* < 0.001). Finally, participants viewed spreading joy and hope as a more fundamental, or core, benefit of the letter-writing task (M_joy_treatment_ = 4.22) than gaining letter-writing practice (M_practice_control_ = 2.32, *t*(98) = 11.70, *P* < 0.001). All pretest responses were on a scale from 1 (Not at all) to 5 (Very much so).

^#^We conducted two rounds of data analysis for this experiment. We first collected 5,938 participants and checked our data. Our results were directional, but not significant. At that point, we submitted a modified preregistration, stipulating that we would add about 12,030 participants and adjust our thresholds of significance to account for the two rounds of data collection. Specifically, we preregistered that we would use the O’Brien–Fleming adjustment rule to reduce our final threshold of significance from *P* = 0.05 to *P* = 0.0471 to account for checking our data at *n* = 5,938. The O’Brien–Fleming adjustment rule is described in more detail in footnote ††. We did not check our data again until data collection was complete. In our *SI Appendix*, we show the results of our preregistered analyses for both rounds of data collection considered separately.

^||^Failure to take any action in response to the email invitation led subjects to donate their earnings, which is why baseline rates of donation are high.

**We focused on the interaction term to test our hypotheses about effort because we expected that, although our treatment would be more effective for frequent gym visitors, this treatment effect would not be large enough to dominate economic incentives.

^††^We consider *P* = 0.049 to be marginally significant based on our adjusted *P* value for sequential analysis, which was *P* = 0.0471 as per the O’Brien–Fleming adjustment rules. Sequential analysis is an analysis method that is frequently used in clinical trials to determine stopping rules that control the type 1 error rate while allowing experimenters to check data partway through analysis (e.g., ref. 43). These rules determine new alpha thresholds for determining significance based on how often and when the data are checked. The intuition is that you “spend” part of your alpha each time you check the data, and the ultimate “alpha” spent adds up to a significance level of 0.05. We preregistered that we would use the O’Brien–Fleming adjustment rule, and we checked our data once at *n* = 5,938, about 33% of the way through the study. This means that our adjusted alpha value for determining significance is 0.0471, which means that our *P* value of 0.049 is marginal rather than significant.

^‡‡^It is worth noting that the interaction between treatment and effort in study 2 is no longer significant when we deviate from our preregistered analysis plan and run logit or probit models as robustness checks (both *P*s > 0.1).

^§§^Although we cannot rule out this alternative explanation for this interaction in study 2, it can be ruled out in study 1 because the incentives participants gave up in study 1 were not donated back to the letter-writing campaign. Thus, giving up earnings in study 1 could only deliver self-signaling benefits.

^¶¶^The interaction between treatment and time delay between program completion and email receipt in study 2 holds when we deviate from our preregistered analysis plan and run logit and probit models (*P* = 0.039 and *P* = 0.042, respectively).

^##^Our results suggest that highlighting the intrinsic benefits of incentivized actions, as in our treatment conditions in study 1 and study 2, will increase motivation laundering; however, our treatment is not necessary for motivation laundering to occur. The desire to signal pure motives may be salient for some even when the intrinsic rewards of their actions were not highlighted. Indeed, our study 1 data demonstrate that, even when no intrinsic benefits are mentioned, over one-fourth of participants choose to forgo some or all of their incentives.
