# Supplementary material for: Forgoing earned incentives to signal pure motives
Source: Proc Natl Acad Sci U S A. 2020 Jul 6;117(29):16891–7. doi: 10.1073/pnas.2000065117 (PMC7382217; doi:10.1073/pnas.2000065117)
Supplement: Supplementary File [file pnas.2000065117.sapp.pdf]

# Supplementary Information for "Forgoing Earned Incentives to Signal Pure Motives"

## Table of Contents


***Study 1 Additional Details*** .......... **2**

*Preregistered Exploratory Analyses: Testing the Effect of Our Treatment on Self-Reported Authentic Motivation* .......... 2

*Preregistered Exploratory Analyses: Testing Letter Quality as a Moderator of Forgone Incentives* .......... 2

*Re-Analyzing Our Results with a Binary Rather than Continuous DV for Forgoing Any Incentive* .......... 3

*Robustness of the Interaction between Time Spent Composing a Letter and Assignment to Our Treatment Condition* .......... 4

***Study 2 Additional Details*** .......... **6**

*Re-Analyzing Our Results for the First 5,938 and Last 12,030 Participants Separately* .......... 6

*Results for First 5,938 Participants Alone* .......... 6

*Results for Last 12,030 Participants Alone* .......... 6

***Study S1: Online Experiment.*** .......... **8**

***Figure S1: Distribution of Time Participants Spent Writing Letters in Study 1*** .......... **13**

***Figure S2: Distribution of Time Delay Between StepUp Program Completion and Email Receipt in Study 2*** .......... **14**

***Figure S3: Study 2 Regression-Estimated Treatment Effects by Gym Visit Tercile*** .......... **15**

***Figure S4: Percent of Study 2 Participants Donating Incentives by Experimental Condition Across Quintiles of Gym Visit Frequency*** .......... **16**

***Figure S5: Study S1 Incentives Forgone by Experimental Condition and Authentic Prosociality Score*** .......... **17**

***Table S1: Regression-Estimated Effects of Experimental Condition on Forgone Incentives in Study 1*** .......... **18**

***Table S2: Regression-Estimated Effects of Treatment Condition on Donating Incentives in Study 2*** .......... **19**

***Table S3: Regression-Estimated Effects of the Interaction Between Gym Visits and Treatment Condition on Donating Incentives in Study 2*** .......... **20**

***Table S4: Regression-Estimated Effects of the Interaction Between Treatment Condition and Time Delay on Donating Incentives in Study 2*** .......... **21**

***Screenshots of Study 1 Stimuli*** .......... **22**

***Screenshots of Study 2 Stimuli*** .......... **35**

***Screenshots of Study S1 Stimuli*** .......... **38**


www.pnas.org/cgi/doi/10.1073/pnas.2000065117

**Study 1 Additional Details**

***Preregistered Exploratory Analyses: Testing the Effect of Our Treatment on Self-Reported Authentic Motivation***

After participants decided how much of their bonus to keep and how much to forgo, we asked them to rate their agreement with a preregistered two-item scale: "I engaged in the letter writing task primarily to help a child in need" and "My decision to write a letter to a child in need was authentic." For both items, participants rated their agreement on a scale from 1 (Not at all) to 7 (Extremely). The two items were averaged to measure self-reported motive authenticity for the letter writing task. We preregistered an ordinary least squares regression predicting self-reported motive authenticity with indicators for experimental condition (baseline control indicator omitted).

There was no significant difference in self-reported motive authenticity across the three experimental conditions. Participants in the treatment condition self-reported an average level of motive authenticity of 5.74, which was not significantly different than the average self-reported level of motive authenticity in the control condition (5.68; $p = .630$). Wald tests confirmed that there was no significant difference in the self-reported motive authenticity between the treatment and active control condition, either (5.69; $p = .722$), nor between the two control conditions ($p = .901$).

***Preregistered Exploratory Analyses: Testing Letter Quality as a Moderator of Forgone Incentives***

We tested whether participants who wrote higher-quality letters, as assessed by their peers, were more willing to forgo some or all of their earnings in the treatment condition. Here, letter quality was intended to serve as a proxy for effort put forth on the incentivized task, albeit a noisy proxy given that letter quality may correlate more strongly with writing skills than effort.

To assess letter quality, we asked a group of 763 MTurk workers to rate the letters written by Study 1 participants. Each MTurk worker rated three randomly selected letters, and each letter was rated by three MTurk workers. For each letter assessed, MTurk workers responded to the question, "How would you rate the overall quality of this letter?" from 1 (Extremely poor) to 7 (Absolutely excellent). We averaged these three ratings to create a quality score for each letter and mean-centered the scores across our sample. We ran an ordinary least squares regression predicting incentives forgone with the following predictor variables: indicators for our treatment condition and our active control condition (the baseline control condition was omitted), our mean-centered measure of letter quality generated by MTurk ratings, and an interaction between each experimental condition indicator and our letter quality measure. The interaction between letter quality and our treatment condition was not significant ($b = 0.025$, SE = 0.060, $p = .672$).

***Re-Analyzing Our Results with a Binary Rather than Continuous DV for Forgoing Any Incentive***

In our main manuscript, we present the results of ordinary least squares regressions with the amount of incentive money forgone as our dependent variable for each of our main analyses in Study 1. As a preregistered robustness check, we also ran both of our main analyses using ordinary least squares regressions with robust standard errors where the dependent variable was a binary indicator for whether a participant decided to forgo any of their incentive money.

First, we ran an ordinary least squares regression with robust standard errors with an indicator for whether a participant was in the treatment condition and an indicator for whether a participant was in the active control condition (with the baseline control condition as the reference group) to determine whether participants in the treatment condition were more likely to forgo some or all of their incentive money. Participants assigned to our treatment condition were significantly more likely to forgo some or all of their earnings: There was a 49.8% increase ($b$ =

0.136, $p$ = .001) in the rate of forgoing incentives in the treatment condition (40.6%) relative to the baseline control condition (27.1%). A Wald test confirmed that assignment to the treatment condition also significantly increased participants' willingness to forgo their earnings in Study 1 by 40.0% relative to the active control condition (percent forgoing incentives in active control = 29.0%; $b$ = 0.117, $p$ = .006), and the difference between the active and baseline control conditions was not significant ($p$ = .633). These results are consistent with the Study 1 findings reported in our main text, and they support our prediction that people are more willing to forgo incentives when reminded of the intrinsic rewards of their actions.

***Robustness of the Interaction between Time Spent Composing a Letter and Assignment to Our Treatment Condition***

In our main manuscript, we present the results of an ordinary least squares regression where we predict Study 1 participants' forgone incentives with a mean-centered measure of the time participants spent composing their letters (in seconds), indicators for their experimental condition (with the baseline control condition omitted), and the interaction between each experimental condition indicator and time spent composing their letter. We found our treatment effect was stronger among participants who had spent more time crafting their letters. The time spent writing letters was heavily right-skewed, however ($M$ = 265.3, $SD$ = 260.4; see Figure S1 for the distribution of letter writing times). As a robustness check, we tested whether the results from our main manuscript remained consistent when we winsorized this skewed measure of effort at various thresholds. Specifically, we winsorized this effort measure at the 95$^{th}$, 97.5$^{th}$, and 99$^{th}$ percentiles (776.7 seconds, 1042.63 seconds, and 1391.62 seconds, respectively). We chose to begin winsorizing at the 95$^{th}$ percentile rather than the 90$^{th}$ percentile because the 90$^{th}$ percentile (532.3 seconds) was within one standard deviation of the mean letter writing time.

When winsorizing at the 95th percentile, we found a significant and positive interaction between time spent crafting a letter and assignment to our treatment condition (*b* = 0.137, SE = 0.0655, *p* = .037) such that an extra 260 seconds spent on letter writing was associated with an 84% increase in the size of the treatment effect. When winsorizing at the 97.5th percentile, these results remained consistent: a one standard deviation increase in time spent on task was associated with an 81.5% increase in the treatment effect (*b* = 0.132, SE = 0.0626, *p* = .035). Finally, our interaction held when winsorizing at the 99th percentile as well (*b* = 0.124, SE = 0.0556, *p* = .026), where the results suggested that a one standard deviation increase in letter writing time was associated with an 76.5% increase in the treatment effect. Overall, these results demonstrate the robustness of the interaction between effort on the incentivized task, as measured by time spent crafting the letter, and our treatment.

## Study 2 Additional Details

### ***Re-Analyzing Our Results for the First 5,938 and Last 12,030 Participants Separately***

As we mention in Footnote 5 in our manuscript, we conducted two rounds of data analysis for this experiment. The first round included 5,938 participants, and the second included 12,030 participants. As a robustness check, we also ran our analyses for each of these two datasets separately.

*Results for First 5,938 Participants Alone*

First, we ran our preregistered ordinary least squared regression with robust standard errors to predict the effect of treatment on donating incentives. We found that participants were directionally more likely to donate their earnings in the treatment condition than in the control condition (estimated increase in likelihood of donating earnings: 1.38 percentage points, 95% CI [-0.179, 2.95]; $p = .083$). We also tested our moderation hypothesis on this smaller dataset. We found that the treatment effect was larger for participants who had exercised more during the StepUp program (estimated increase in effectiveness of treatment for each added gym visit during the incentive program: 2.02 percentage points, 95% CI [0.091, 3.94]; $p = .040$).

*Results for Last 12,030 Participants Alone*

Next, we ran our preregistered analyses on the second set of 12,030 participants. We first tested our main effect of treatment on donating incentives. We found that participants in the treatment condition were directionally more likely to donate their earnings than those in the control condition (estimated increase in likelihood of donating earnings: 0.899 percentage points, 95% CI [-0.0226, 1.82]; $p = .056$). We then examined whether our treatment was more effective for participants who exercised more frequently. We found that on this subset of the data, exercise frequency did not significantly moderate the effect of treatment on willingness to donate

incentives, though our treatment effect was directionally larger for participants who had exercised more during the StepUp program (estimated increase in effectiveness of treatment for each added gym visit during the incentive program: 0.670 percentage points, 95% CI [-0.546, 1.89]; $p = .280$).

**Study S1: Online Experiment.**

***Method.*** This experiment followed the design of Study 1 in the main manuscript. Four hundred and sixty-five participants were recruited through Amazon's Mechanical Turk to participate in a preregistered[1] study in exchange for $0.30. All participants were asked to complete a short image classification task irrelevant to the remainder of the study. They were then offered the choice to opt in to complete a second task for a $2.00 bonus. The 454 participants who opted to complete the second task (50.4% female) were randomly assigned to a condition and included in our analysis, following our preregistration.

Participants were asked to write a letter to provide hope to a sick child who would be hospitalized during the upcoming holiday season. All letters deemed appropriate were actually sent to sick children via the I See Me! Letters of Love campaign in December 2019, in partnership with the Children's Cancer Research Fund.

To measure the value congruence of the intrinsic reward, we adapted the authentic prosociality scale (1). In all conditions, participants responded to the authentic prosociality scale items after writing their letters to hospitalized children but before deciding how much of their bonus to keep and how much to forgo. Participants rated their agreement on a scale from "1 – Not at all" to "7 – Extremely" with the following five items: "I sincerely care about helping children in need"; "I engaged in the letter writing task primarily to help children in need"; "I engaged in the letter writing task primarily to earn bonus money" (reverse scored); "I have a genuine passion for helping children who are in the hospital this holiday season"; and "My decision to write a letter to a child in need was authentic." Participants rated agreement with each item on a 1-7 scale. The mean level of authentic prosociality was 5.41, and the standard

---

[1] http://aspredicted.org/blind.php?x=ek5xn5

deviation was 1.29. We used this scale to measure the value congruence of the emphasized intrinsic benefits of the letter writing activity.

After participants completed this measure, they were randomized into one of three experimental conditions: a treatment condition, an active control condition, and a baseline control condition. In all conditions, participants were given the opportunity to forgo some or all of their $2.00 earnings from writing the letter. In the treatment condition, we emphasized the intrinsic reward of the letter writing activity by prompting them to "treat the joy and hope you've spread as your reward" before inviting them to forgo some or all of their bonus. In the active control condition, we emphasized a less-valued reward of the letter writing activity by prompting participants to "treat the letter writing practice you've received as your reward" before inviting them to forgo some or all of their bonus. As in Study 1 in the main manuscript, this active control condition allows us to test our hypothesis that participants will be more likely to forgo their incentives when they are prompted with value-congruent intrinsic benefits rather than value-incongruent intrinsic benefits. In the baseline control condition, participants did not receive any additional prompting before being invited to forgo some or all of their bonus.

Finally, we collected two exploratory measures after participants had decided how much of their bonus to forgo. Participants rated their agreement with each of these items on a scale from 1 (Not at all) to 7 (Extremely). The first item measured feelings of guilt ("The message I read made me feel guilty about keeping my bonus"), and the second item measured feelings of gratitude ("I felt grateful for the opportunity to participate in the letter drive"). We preregistered exploratory mediation tests to assess whether guilt or gratitude mediated the effect of our treatment on willingness to forgo incentives.

***Results.*** Our dependent variable of interest was the quantity of a participant's forgone earnings (a value that could range from 0 to 200 cents). Participants gave up 29.3 cents on average (s.d. = 59.9) in the treatment condition, 13.0 cents on average (s.d. = 38.6) in the active control condition, and 12.7 cents (s.d. = 39.9) on average in the baseline control condition. We found that 22.4% of participants gave up some or all of their incentives in the treatment condition, while only 12.0% chose to forgo any earnings in the active control condition and 12.5% chose to forgo any earnings in the baseline control condition.

Following our preregistered analysis plan, we ran an ordinary least squares regression to predict each participant's forgone earnings. The only predictor variables in this regression were indicators for experimental conditions, and the indicator for our baseline control condition was omitted. Assignment to our treatment condition significantly increased forgone earnings by \$0.17 (or 131%, $p = .0023$) relative to assignment to the baseline control condition. A Wald test confirmed that assignment to the treatment condition also significantly increased forgone earnings by \$0.16 (or 125%, $p = .0029$) relative to assignment to the active control condition, and the difference between being in the active and baseline control conditions was not significant ($p = .95$).

Thus, we replicated our findings from Study 1 in the main manuscript: people were more likely to forgo earned incentives when reminded of the intrinsic, non-monetary rewards of their actions. As in Study 1, participants were most likely to forgo their incentives when the intrinsic rewards emphasized would promote a positive self-image, suggesting willingness to give up earnings is driven by self-image concerns.

We also examined whether participants who felt that the non-monetary rewards of the letter writing exercise were value-congruent responded more strongly to our treatment. That is,

we examined whether those who scored high on authentic prosociality (participants who reported that they genuinely cared about the sick children, which motivated their decision to write the letter) showed a larger treatment effect. Following our preregistration, we collapsed across the active and baseline control conditions and ran an ordinary least squares regression predicting incentives forgone with three predictor variables: a treatment condition indicator, our mean-centered measure of authentic prosociality, and an interaction between these variables. As hypothesized, the interaction between authentic prosociality and assignment to our treatment condition was significant and positive ($b$ = 0.1215, SE = 0.0442, $p$ = .0063; see Figure S5). Specifically, we found that a one standard deviation increase in authentic prosociality increases the estimated treatment effect by 74%. In short, the more participants felt that the letter-writing task's non-monetary rewards reflected their values, the more likely they were to give up their earnings in the treatment condition.

Finally, we tested our exploratory mediation hypotheses to examine whether guilt and gratitude mediate the effect of treatment on willingness to forgo incentives. Gratitude did not significantly mediate the effect of our treatment on forgoing incentives. There was no significant main effect of assignment to the treatment condition on feelings of gratitude ($b$ = 0.025, $SE$ = 0.136, $p$ = .852), and feelings of gratitude did not significantly predict the amount of incentive money that participants chose to forgo ($b$ = 0.013, $SE$ = 0.012, $p$ = .29). A Sobel test confirmed that there was no significant reduction in effect size when controlling for feelings of gratitude ($b_{reduction}$= 0.0003, $SE$ = 0.002, $p$ = .854), and a 10,000-sample bootstrap analysis (MacKinnon, Fairchild, & Fritz, 2007; Shrout & Bolger, 2002) produced a bias-corrected 95% confidence interval for the size of the indirect effect that included zero (95% CI: [-0.088, 0.014]).

However, guilt did mediate the effect of our treatment on forgoing incentives. First, there was a significant main effect of assignment to the treatment condition on the degree to which participants reported feeling guilty because of the message they read ($b = 0.517$, $SE = 0.199$, $p = .0096$). Second, the relationship between feeling guilty and the amount of their earnings participants chose to forgo was also significant ($b = 0.041$, $SE = 0.011$, $p < .001$). Consistent with mediation, the effect of assignment to the treatment condition on amount of incentive money forgone ($b = 0.164$, $SE = 0.047$, $p < .001$) was reduced when controlling for feelings of guilt ($b = 0.145$, $SE = 0.047$, $p = .002$). A Sobel test confirmed that this reduction in effect size was significant ($b_{reduction} = 0.02$, $SE = 0.009$, $p = .016$), and a 10,000-sample bootstrap analysis produced a bias-corrected 95% confidence interval for the size of the indirect effect that excluded zero (95% CI: [0.003, 0.043]). This suggests that, consistent with prior literature, guilt can motivate self-signaling behavior (2, 3), although the results of Study 1 in the main manuscript suggest that this guilt did not leave participants worse off.

**Figure S1: Distribution of Time Participants Spent Writing Letters in Study 1**

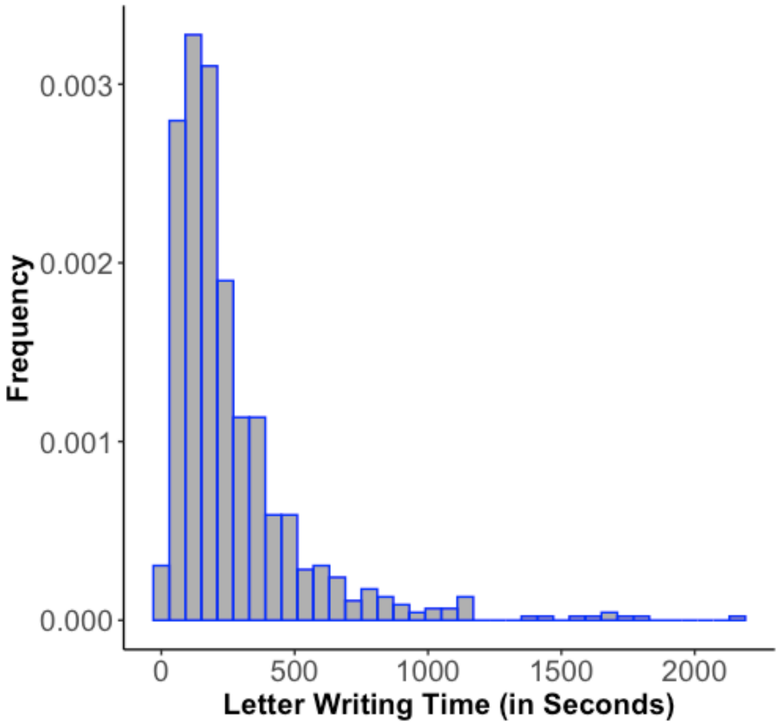


*Figure S1.* This histogram shows the distribution of time participants spent writing letters (in seconds) in Study 1. The mean time spent on this task was 265 seconds, and the standard deviation was 260 seconds. In all regressions including letter writing time as a predictor variable, a mean-centered measure of letter writing time is used.

**Figure S2: Distribution of Time Delay Between StepUp Program Completion and Email Receipt in Study 2**

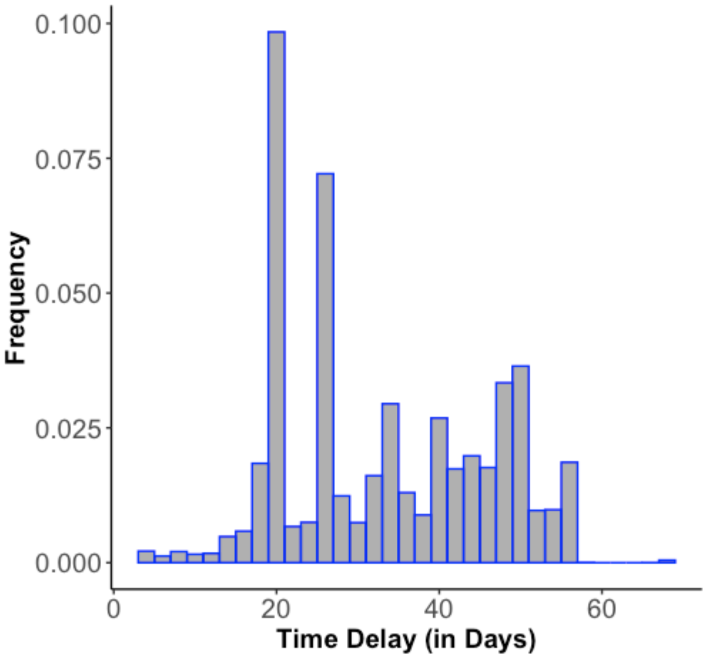


*Figure S2.* This histogram shows the distribution of the time delay between when study participants completed the StepUp program and when they received an email inviting them to forgo their incentives (in days) in Study 2. The mean time delay was 33.8 days, and the standard deviation was 12.6 days. In all regressions including time delay as a predictor variable, a mean-centered measure of time delay is used.

**Figure S3: Study 2 Regression-Estimated Treatment Effects by Gym Visit Tercile**

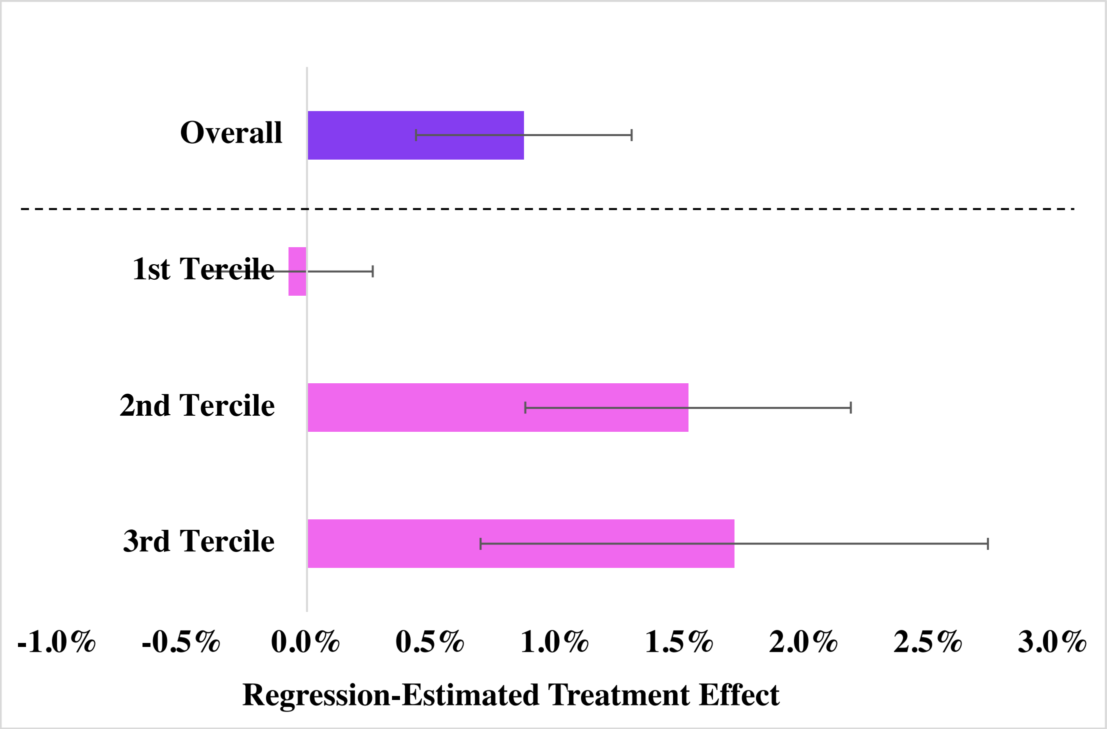


*Figure S3.* The top bar in the figure shows the regression-estimated treatment effect produced by emphasizing intrinsic rewards on a study participant's likelihood of donating their earnings across all participants. Below the dotted line, we depict the regression-estimated treatment effects for participants in the first, second, and third tercile of gym visits (estimated separately). Error bars depict 95% confidence intervals. There is no significant treatment effect for participants in the lowest tercile of gym visits, but a significant treatment effect emerges for participants in the 2nd and 3rd terciles.

**Figure S4: Percent of Study 2 Participants Donating Incentives by Experimental Condition Across Quintiles of Gym Visit Frequency**

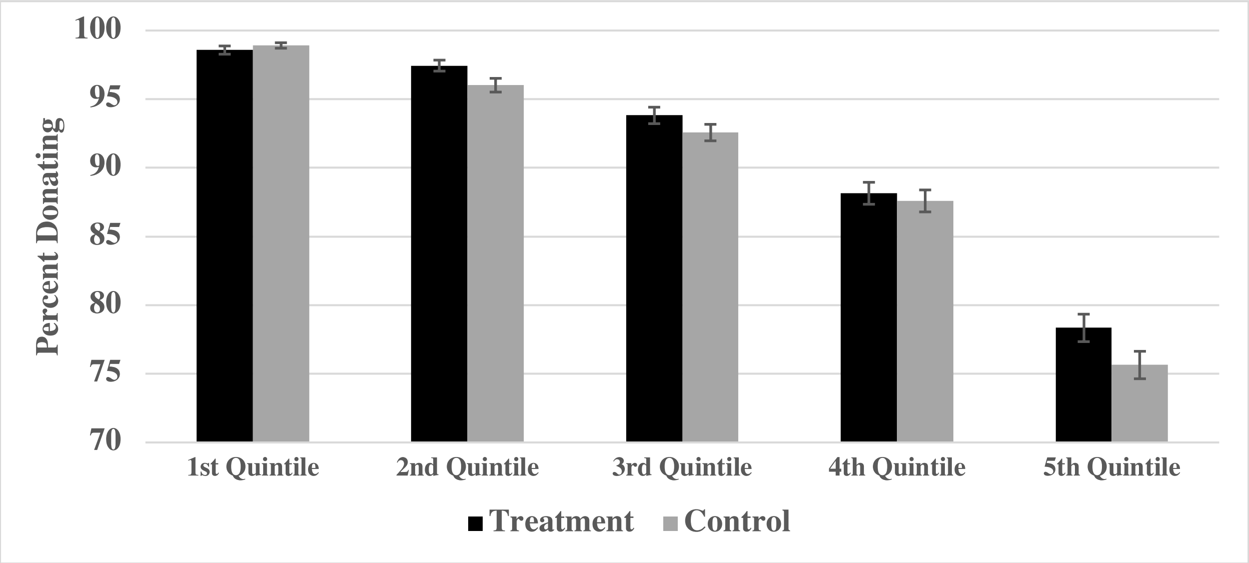


*Figure S4.* This figure depicts the percent of participants in Study 2 donating their incentives across experimental conditions for each of the five quintiles of participant gym visit frequency during the StepUp program. Black bars represent the proportion of participants donating in the treatment condition and grey bars represent the proportion of participants donating in the control condition. Participants in the first quintile visited the gym 0 times during the StepUp program, while those in the fifth quintile visited the gym more than 12 times during the StepUp program. The largest treatment effect is in the 5$^{th}$ quintile of gym visits. Error bars depict +/- 1 standard error.

**Figure S5: Study S1 Incentives Forgone by Experimental Condition and Authentic Prosociality Score**

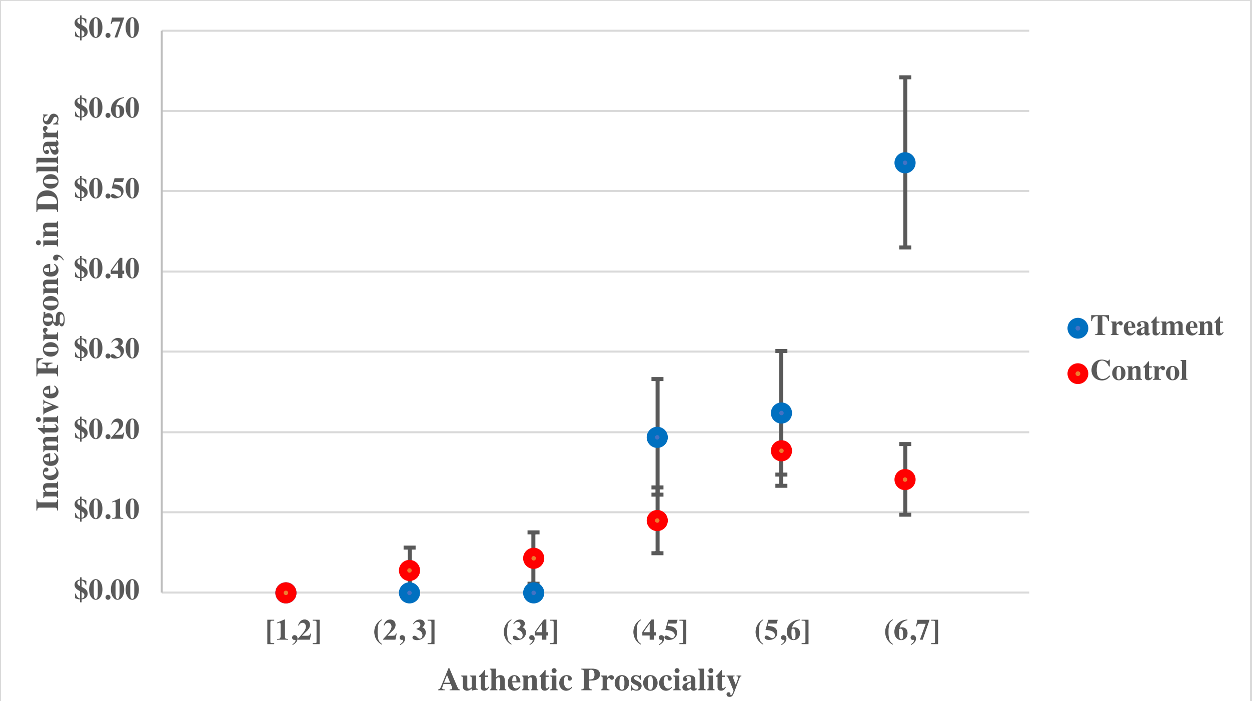


*Figure S5.* The effect of intrinsic reward reminders on the amount forgone in Study S1 was strongest for those who were highest in self-reported authentic prosociality. The figure plots the average dollar amount forgone for Study S1 participants in our treatment group and our two control groups (combined) within each bin of self-reported authentic prosociality. Standard error bars are included around each mean but are missing for authentic prosociality bin [1,2] for both conditions and (2,3] for the treatment condition because all participants within those bins and conditions gave up $0.00.

**Table S1: Regression-Estimated Effects of Experimental Condition on Forgone Incentives in Study 1**

| | How Much of the Incentive Was Forgone? (In Dollars) |
|---|---|
| | **Model 1** |
| *Treatment* | 0.162** (0.057) |
| *Active Control* | 0.043 (0.057) |
| Wald Test: Difference between Coefficients on Treatment and Active Control Indicators | 0.119* (0.057) |
| **Observations** | 763 |
| **Adjusted $R^2$** | 0.009 |

*Table S1*. This table reports the results of an ordinary least squares (OLS) regression predicting how much money participants in Study 1 chose to forgo (from $0 to $2) with an indicator for whether they were assigned to our treatment condition and an indicator for whether they were assigned to our active control condition. A Wald test compares the effect of assignment to the treatment condition with the effect of assignment to the active control condition. Robust standard errors are in parentheses.

†, *, **, and *** denote significance at the 10%, 5%, 1%, and 0.1% levels, respectively.

**Table S2: Regression-Estimated Effects of Treatment Condition on Donating Incentives in Study 2**

| | Was the Incentive Donated? (1 = Yes, 0 = No) | | | |
|---|---|---|---|---|
| | **Model 1** | **Model 2** | **Model 3** | **Model 4** |
| *Treatment* | 0.009*<br>(0.004) | 0.007†<br>(0.004) | 0.010*<br>(0.004) | 0.010*<br>(0.004) |
| *Cash Earned in StepUp* | | | -0.029***<br>(0.001) | -0.029***<br>(0.001) |
| *Male* | | | | 0.009*<br>(0.004) |
| *Sex Unknown* | | | | -0.011<br>(0.017) |
| *Fixed Effects for Version of StepUp Program Participant Experienced* | No | Yes | Yes | Yes |
| **Observations** | 17,968 | 17,968 | 17,968 | 17,968 |
| **Adjusted $R^2$** | 0.0001 | 0.025 | 0.110 | 0.111 |

*Table S2*. This table reports the results of four ordinary least squares (OLS) regression models predicting whether a StepUp participant in Study 2 chose to donate their incentives. The only predictors in Model 1 is an indicator for assignment to our treatment condition. Model 2 adds fixed effects for which version of the StepUp program the participant experienced. Model 3 adds a control for the amount of cash earned by the participant in StepUp. Model 4 adds indicators for available information about the participant's sex (an indicator for whether the participant reported that they were male and an indicator for sex unknown). Robust standard errors are in parentheses.

†, *, **, and *** denote significance at the 10%, 5%, 1%, and 0.1% levels, respectively.

**Table S3: Regression-Estimated Effects of the Interaction Between Gym Visits and Treatment Condition on Donating Incentives in Study 2**

| | **Was the Incentive Donated? (1 = Yes, 0 = No)** | | | |
|---|---|---|---|---|
| | **Model 1** | **Model 2** | **Model 3** | **Model 4** |
| *Treatment* | 0.012**<br>(0.003) | 0.010*<br>(0.004) | 0.011**<br>(0.004) | 0.011**<br>(0.004) |
| *Number of Gym Visits (during StepUp)* | -0.089***<br>(0.004) | -0.091***<br>(0.004) | -0.058***<br>(0.004) | -0.059***<br>(0.004) |
| *Treatment*Number of Gym Visits* | 0.009†<br>(0.005) | 0.011*<br>(0.005) | 0.010*<br>(0.005) | 0.010*<br>(0.005) |
| *Cash Earned in StepUp* | | | -0.017***<br>(0.001) | -0.017***<br>(0.001) |
| *Male* | | | | 0.020***<br>(0.004) |
| *Sex Unknown* | | | | -0.010<br>(0.016) |
| *Fixed Effects for Version of StepUp Program Participant Experienced* | No | Yes | Yes | Yes |
| **Observations** | 17,968 | 17,968 | 17,968 | 17,968 |
| **Adjusted $R^2$** | 0.084 | 0.111 | 0.130 | 0.131 |

*Table S3*. This table reports the results of four ordinary least squares (OLS) regression models predicting whether a StepUp participant in Study 2 chose to donate their incentives. Predictors in Model 1 include an indicator for the treatment condition, the participants' mean-centered number of gym visits during the incentive period, and the interaction between gym visits and assignment to the treatment condition. Model 2 adds fixed effects for which version of the StepUp program the participant experienced. Model 3 adds the amount of cash earned by the participant in the StepUp program. Model 4 adds indicators for available information about the participant's sex (an indicator for whether the participant reported that they were male and an indicator for sex unknown). Robust standard errors are in parentheses.
†, *, **, and *** denote significance at the 10%, 5%, 1%, and 0.1% levels, respectively.

**Table S4: Regression-Estimated Effects of the Interaction Between Treatment Condition and Time Delay on Donating Incentives in Study 2**

| | Was the Incentive Donated? (1 = Yes, 0 = No) | | | |
|---|---|---|---|---|
| | **Model 1** | **Model 2** | **Model 3** | **Model 4** |
| *Treatment* | 0.034**<br>(0.013) | 0.031*<br>(0.012) | 0.035**<br>(0.012) | 0.035**<br>(0.012) |
| *Time Delay Between Program Completion and Email Receipt (in Days)* | 0.001***<br>(0.0002) | 0.0006***<br>(0.0002) | 0.0009***<br>(0.0003) | 0.0009***<br>(0.0003) |
| *Treatment*Time Delay (in Days)* | -0.0007*<br>(0.0003) | -0.0007*<br>(0.0003) | -0.0007*<br>(0.0003) | -0.0007*<br>(0.0003) |
| *Cash Earned in StepUp* | | | -0.029*<br>(0.001) | -0.029***<br>(0.001) |
| *Male* | | | | 0.009*<br>(0.004) |
| *Sex Unknown* | | | | -0.010<br>(0.016) |
| *Fixed Effects for Version of StepUp Program Participant Experienced* | No | Yes | Yes | Yes |
| **Observations** | 17,968 | 17,968 | 17,968 | 17,968 |
| **Adjusted $R^2$** | 0.001 | 0.026 | 0.111 | 0.111 |

*Table S3*. This table reports the results of four ordinary least squares (OLS) regression models predicting whether a StepUp participant in our experiment chose to donate their incentives in Study 2. Model 1 shows moderation without controlling for any covariates, while Models 2-4 incrementally add covariates. Predictors in Model 1 include an indicator for assignment to the treatment condition, a mean-centered continuous variable representing the time delay (in days) between program completion and email receipt, and the interaction between time delay and assignment to the treatment condition. Model 2 adds fixed effects for which version of the StepUp program the participant experienced. Model 3 adds the amount of cash earned by the participant in the StepUp program. Model 4 adds indicators for available information about the participant's sex (an indicator for whether the participant reported that they were male and an indicator for sex unknown). Robust standard errors are in parentheses.
†, *, **, and *** denote significance at the 10%, 5%, 1%, and 0.1% levels, respectively.

**Screenshots of Study 1 Stimuli**

*Screen 1 (All experimental conditions):*

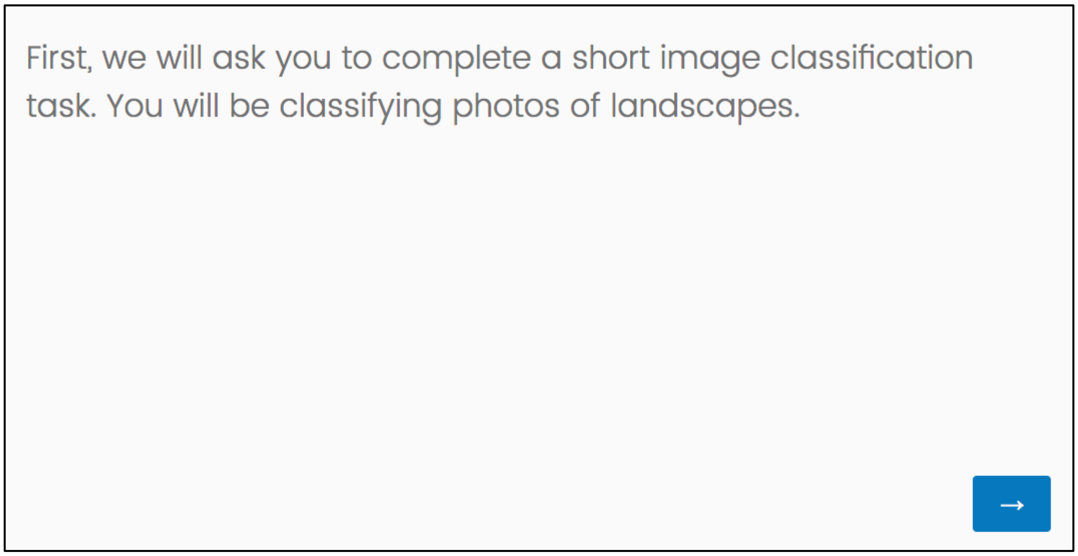


*Screen 2 (All experimental conditions):*

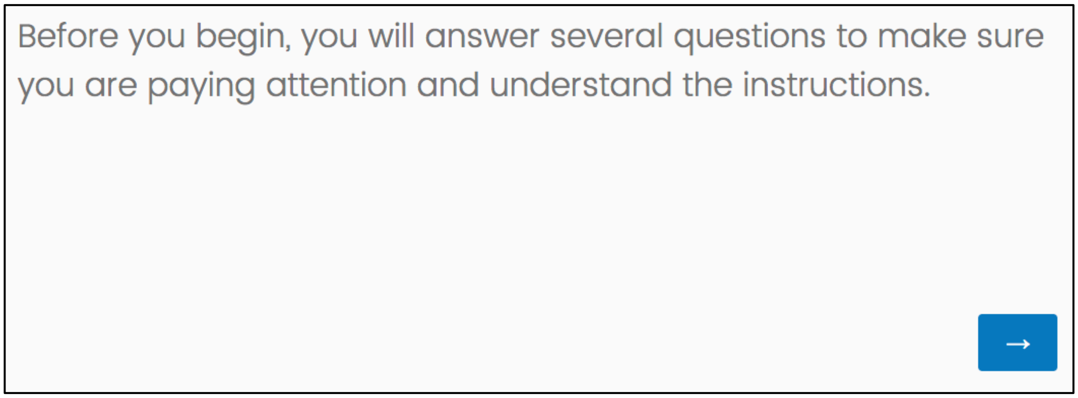

*Screen 3 (All experimental conditions):*

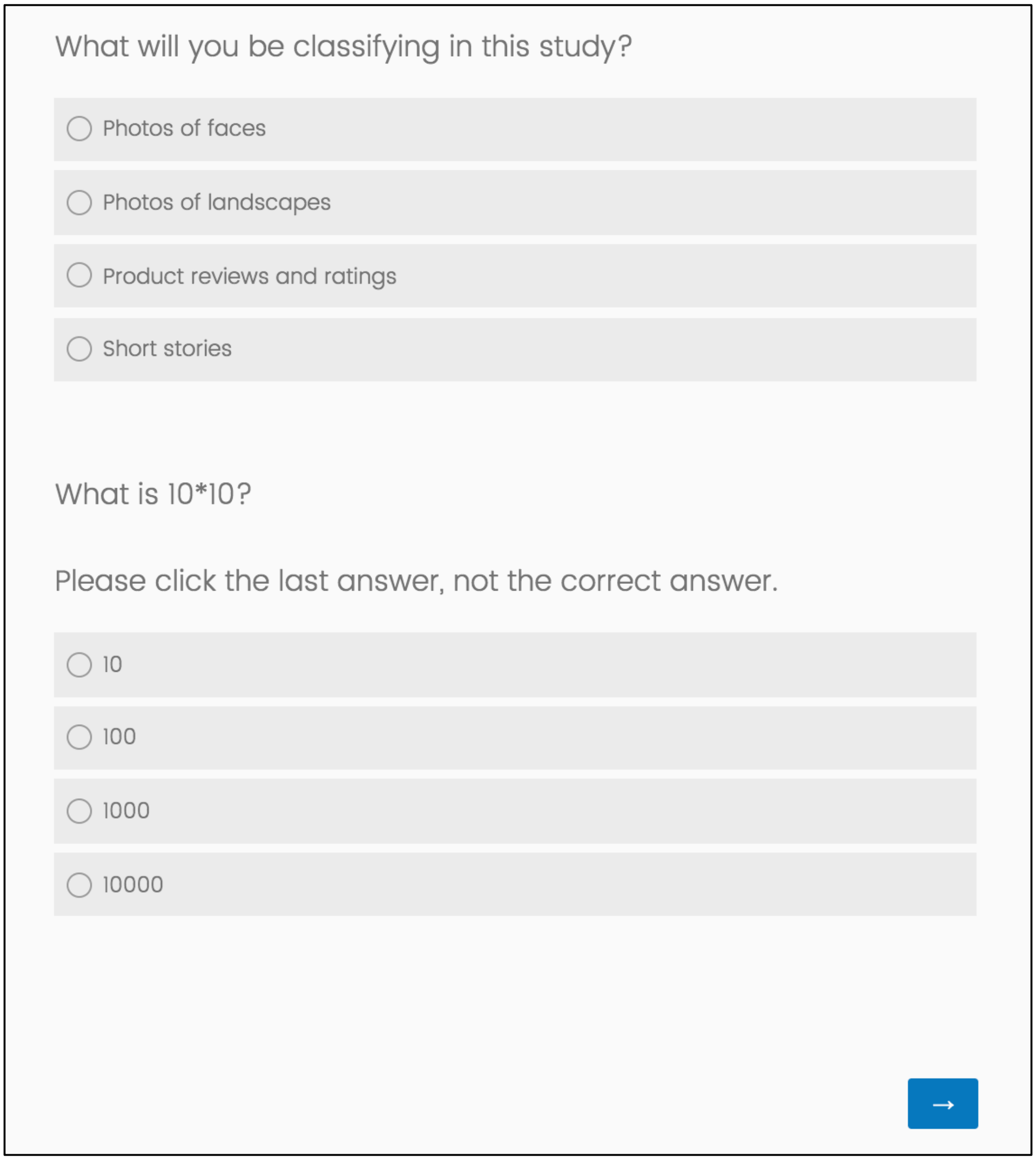
What will you be classifying in this study?

- Photos of faces
- Photos of landscapes
- Product reviews and ratings
- Short stories

What is 10*10?

Please click the last answer, not the correct answer.

- 10
- 100
- 1000
- 10000

→

*Screen 4 (All experimental conditions):*

**Landscape Classification Task:**

You will now look at a series of landscapes and a set of categories that might correspond to the landscape. Please pick the category from the dropdown list that best corresponds to the photo, trying to be as accurate as possible.

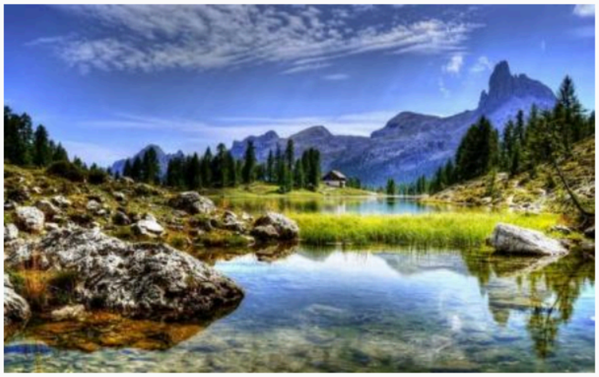

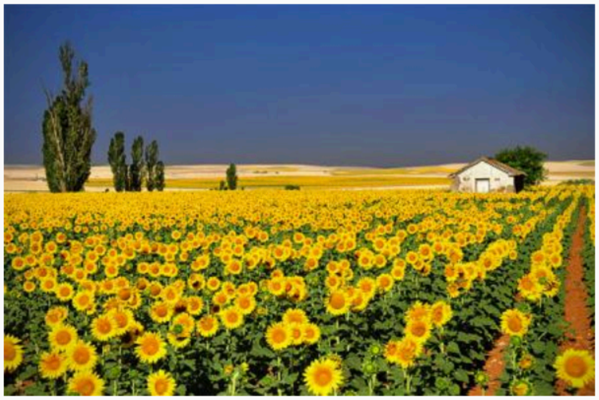

*Screen 5 (All experimental conditions):*

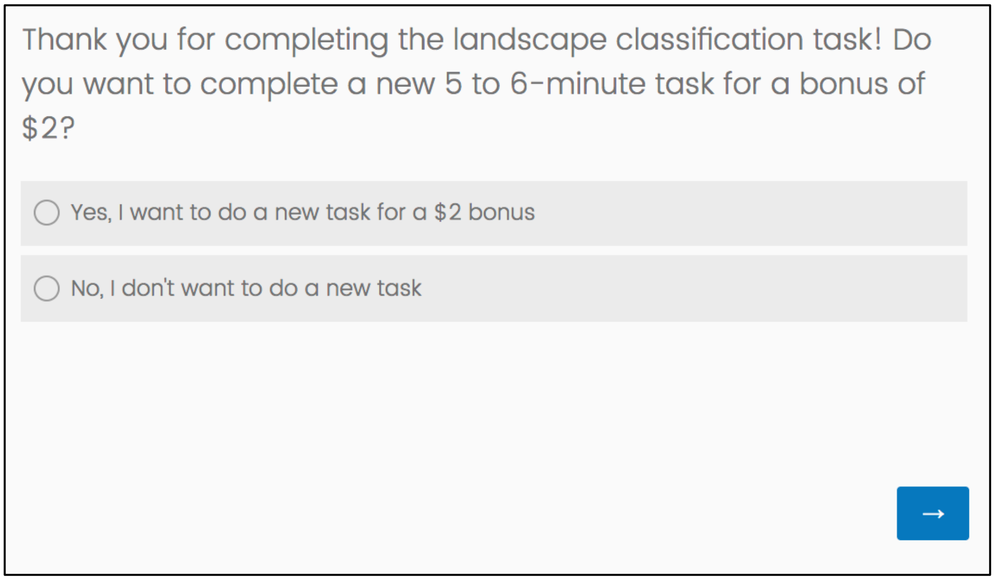


*If participants consent to doing task two, the survey continues.*

*Screen 6 (All experimental conditions):*

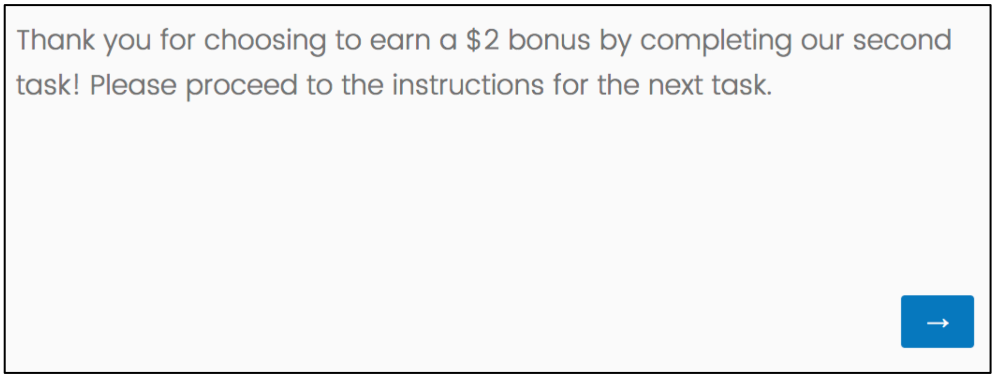

*Screen 7 (All experimental conditions):*

The research team associated with this study is currently running a letter drive to spread good cheer during the holiday season. Specifically, we are partnering with an organization to distribute letters to children who will be spending their holiday season in the hospital. The goal of each letter is to provide hope to a child, and remind the child that others are thinking of them. We are collecting letters ahead of time to make sure we have enough letters when the holidays come around.

We are paying each participant $2 to write a letter to a child, which we will distribute during the upcoming holiday season. Please note that letters will be selected to share with children only if they are deemed appropriate. Keep in mind that letters should be upbeat and positive.

→

*Screen 8 (All experimental conditions):*

Please answer the following questions to ensure you understood the instructions.

→

*Screen 9 (All experimental conditions):*

What is the purpose of the letter drive associated with this study?

- ◯ Spreading good cheer to children in need during the holidays
- ◯ Sending surprise holiday letters to strangers
- ◯ Partnering with pen pals to foster international understanding
- ◯ Spreading hope to our troops

Who will receive your letter?

- ◯ A member of the army stationed overseas this holiday season
- ◯ A child spending the holiday season in the hospital
- ◯ A random stranger
- ◯ A pen pal overseas

→

*Screen 10 (All experimental conditions):*

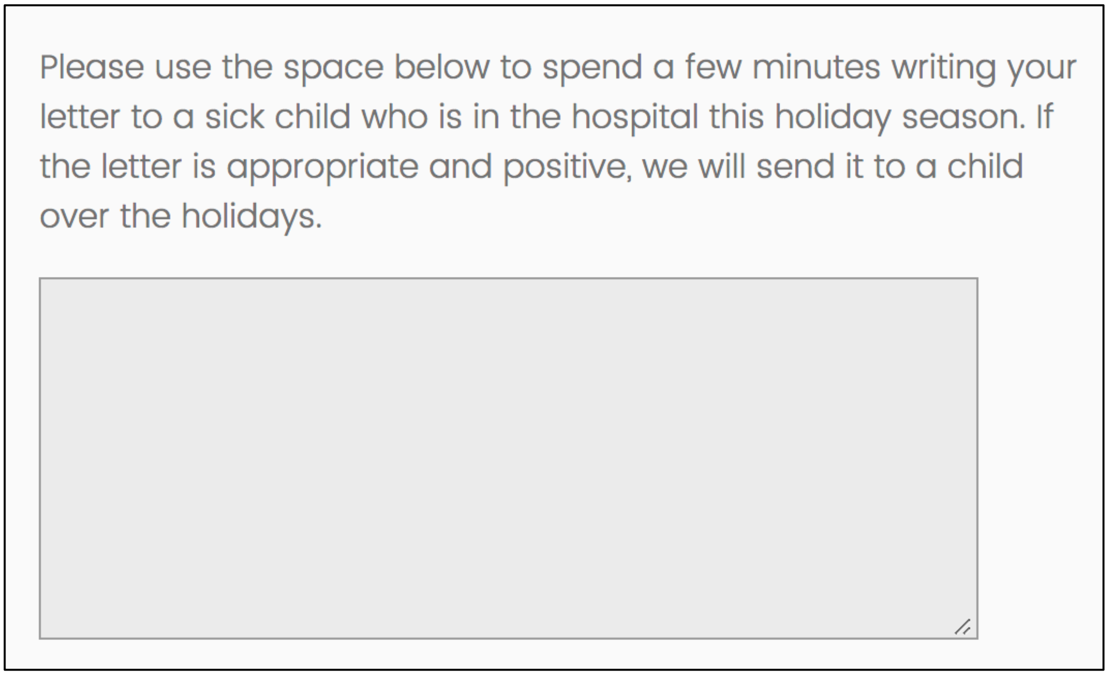
Please use the space below to spend a few minutes writing your letter to a sick child who is in the hospital this holiday season. If the letter is appropriate and positive, we will send it to a child over the holidays.

*Screen 11 (Baseline control condition):*

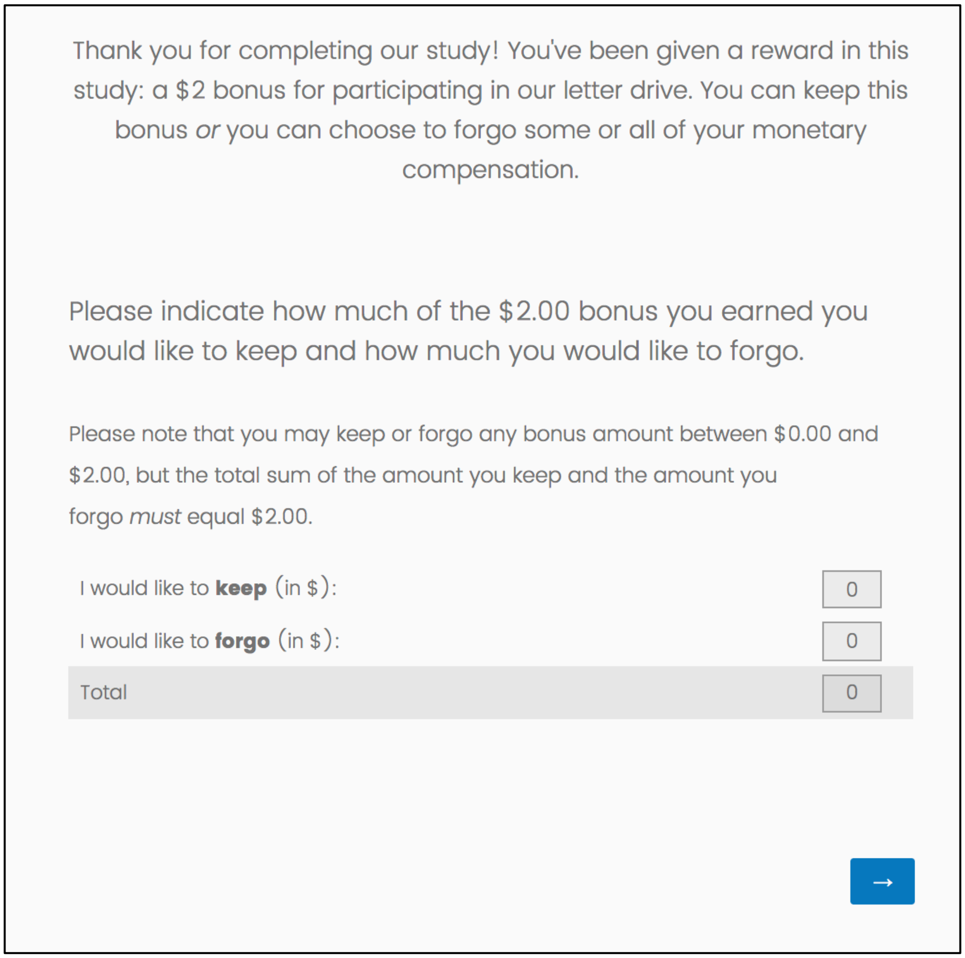
Thank you for completing our study! You've been given a reward in this study: a $2 bonus for participating in our letter drive. You can keep this bonus *or* you can choose to forgo some or all of your monetary compensation.

Please indicate how much of the $2.00 bonus you earned you would like to keep and how much you would like to forgo.

Please note that you may keep or forgo any bonus amount between $0.00 and $2.00, but the total sum of the amount you keep and the amount you forgo *must* equal $2.00.

| | |
|---|---|
| I would like to **keep** (in $): | 0 |
| I would like to **forgo** (in $): | 0 |
| Total | 0 |

→

*Screen 11 (Treatment condition):*

Thank you for completing our study! You've been given two rewards in this study: (1) an opportunity to spread joy and hope by writing a thoughtful letter to a child in need and (2) a $2 bonus for participating in our letter drive. You can keep this bonus *or* **you can treat the joy and hope you've spread as your reward** and choose to forgo some or all of your monetary compensation.

Please indicate how much of the $2.00 bonus you earned you would like to keep and how much you would like to forgo.

Please note that you may keep or forgo any bonus amount between $0.00 and $2.00, but the total sum of the amount you keep and the amount you forgo *must* equal $2.00.

| | |
|---|---|
| I would like to **keep** (in $): | 0 |
| I would like to **forgo** (in $): | 0 |
| Total | 0 |

→

*Screen 11 (Active control condition):*

Thank you for completing our study! You've been given two rewards in this study: (1) an opportunity to practice your letter writing skills by writing a thoughtful letter to a child in need and (2) a $2 bonus for participating in our letter drive. You can keep this bonus *or* **you can treat the letter writing practice you've received as your reward** and choose to forgo some or all of your monetary compensation.

Please indicate how much of the $2.00 bonus you earned you would like to keep and how much you would like to forgo.

Please note that you may keep or forgo any bonus amount between $0.00 and $2.00, but the total sum of the amount you keep and the amount you forgo *must* equal $2.00.

| | |
|---|---|
| I would like to **keep** (in $): | 0 |
| I would like to **forgo** (in $): | 0 |
| Total | 0 |

→

*Screen 12 (All experimental conditions):*

You wrote this letter as part of a larger campaign to support and care for children in need. We are always looking for people to write more letters in the future.

Would you be willing to participate in more studies like this one for $1?

If you click "Yes" below, you will be added to a list of MTurkers eligible for studies like this one and may be asked to participate soon.

- Yes
- No

→

*Screen 13 (All experimental conditions):*

Please indicate how much you agree with the following statements. As a reminder, you chose to forgo $1 of your monetary compensation.

My decision to write a letter to a child in need was authentic

| 1 (Not at all) | 2 | 3 | 4 | 5 | 6 | 7 (Extremely) |
|---|---|---|---|---|---|---|
| ○ | ○ | ○ | ○ | ○ | ○ | ○ |

I engaged in the letter writing task primarily to help a child in need

| 1 (Not at all) | 2 | 3 | 4 | 5 | 6 | 7 (Extremely) |
|---|---|---|---|---|---|---|
| ○ | ○ | ○ | ○ | ○ | ○ | ○ |

→

*Screen 14 (All experimental conditions):*

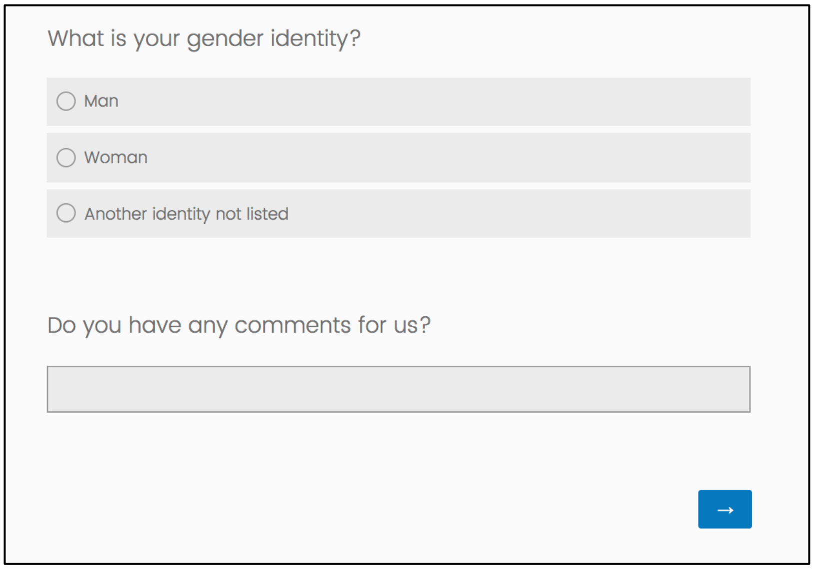
What is your gender identity?

- ◯ Man
- ◯ Woman
- ◯ Another identity not listed

Do you have any comments for us?

→

## Screenshots of Study 2 Stimuli

*Treatment Condition Email Sent to StepUp Participants:*

**Subject:** Redeem or Donate Your StepUp Points

STEP UP

### Redeem or Donate Your StepUp Points

#### Treat the Healthy Habits You've Kick-started As Your Reward and Donate Your Points

StepUp is a not-for-profit program and is funded by universities and foundations, including the [redacted] and the Robert Wood Johnson Foundation.

Congratulations on completing the StepUp Program! You've started building healthy habits, and that's the biggest reward StepUp has to offer! In the process, you've earned 200 StepUp Points. You can redeem these points for a $2.00 Amazon Cash gift card ***or*** **you can treat the healthy habits you've kick-started as your reward and donate these points back to the StepUp program**. (Remember that the conversion rate is 7000 points = $5.00.)

**If you choose to donate your $2.00 back to the StepUp Program, you will be contributing to the continued work of StepUp on the science of healthy habits and helping thousands of others kickstart their healthy habits. Many StepUp participants make this choice to support science.**

If you choose to redeem your points for a gift card, it will be emailed to you within one week, via Tangocard.

**Please click here to tell us what you would like to do with your StepUp points.**

P.S. Congrats again on your earnings and the healthy habits you've kick-started!

Have questions? Contact us at StepUp Support

*Control Condition Email Sent to StepUp Participants:*

Note that the monetary amounts differing across these screenshots is an artifact of testing. In practice, the monetary amounts depended on the participants' incentives and exercise frequency.

**Subject:** Redeem or Donate Your StepUp Points

STEP UP

## Redeem or Donate Your StepUp Points

StepUp is a not-for-profit program and is funded by universities and foundations, including the [redacted] and the Robert Wood Johnson Foundation.

Congratulations on completing the StepUp Program! You've earned 100 StepUp Points. You can redeem these points for a $1.00 Amazon Cash gift card ***or* you can donate these points back to the StepUp program**. (Remember that the conversion rate is 7000 points = $5.00.)

**If you choose to donate your $1.00 back to the StepUp Program, you will be contributing to the continued work of StepUp on the science of healthy habits and helping thousands of others kickstart their healthy habits. Many StepUp participants make this choice to support science.**

If you choose to redeem your points for a gift card, it will be emailed to you within one week, via Tangocard.

**Please click here to tell us what you would like to do with your StepUp points.**

P.S. Congrats again on your earnings!

Have questions? Contact us at StepUp Support

Contact

You are receiving this message as part of your 28-day StepUp Program. Opt out of future emails: Unsubscribe.

*If participants in Study 2 clicked the email where it said "Click Here," they were taken to a Qualtrics survey containing the following question (language in red appeared only in the treatment condition):*

What do you choose to do with your StepUp points?

- [treat the healthy habits you've kick-started as your reward and] give $[X.XX] back to the StepUp Program to support scientific research on healthy habits
- redeem your points for a $[X.XX] Amazon gift card (which will be sent to you by email within one week via TangoCard)

**Screenshots of Study S1 Stimuli**

*Screen 1 (All experimental conditions):*

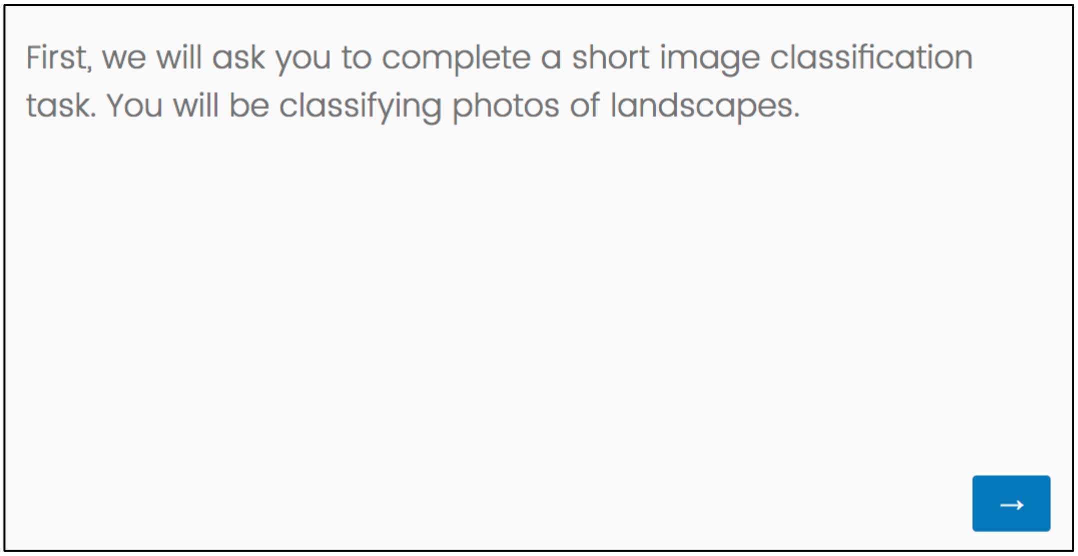


*Screen 2 (All experimental conditions):*

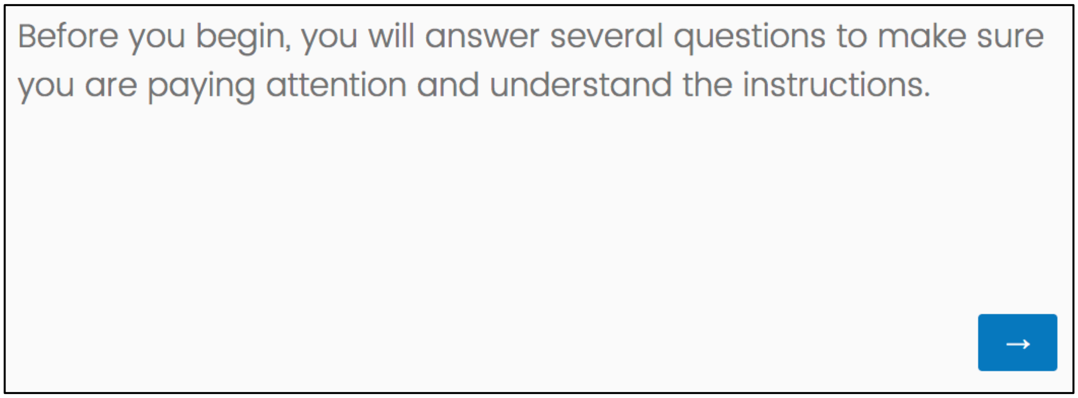

*Screen 3 (All experimental conditions):*

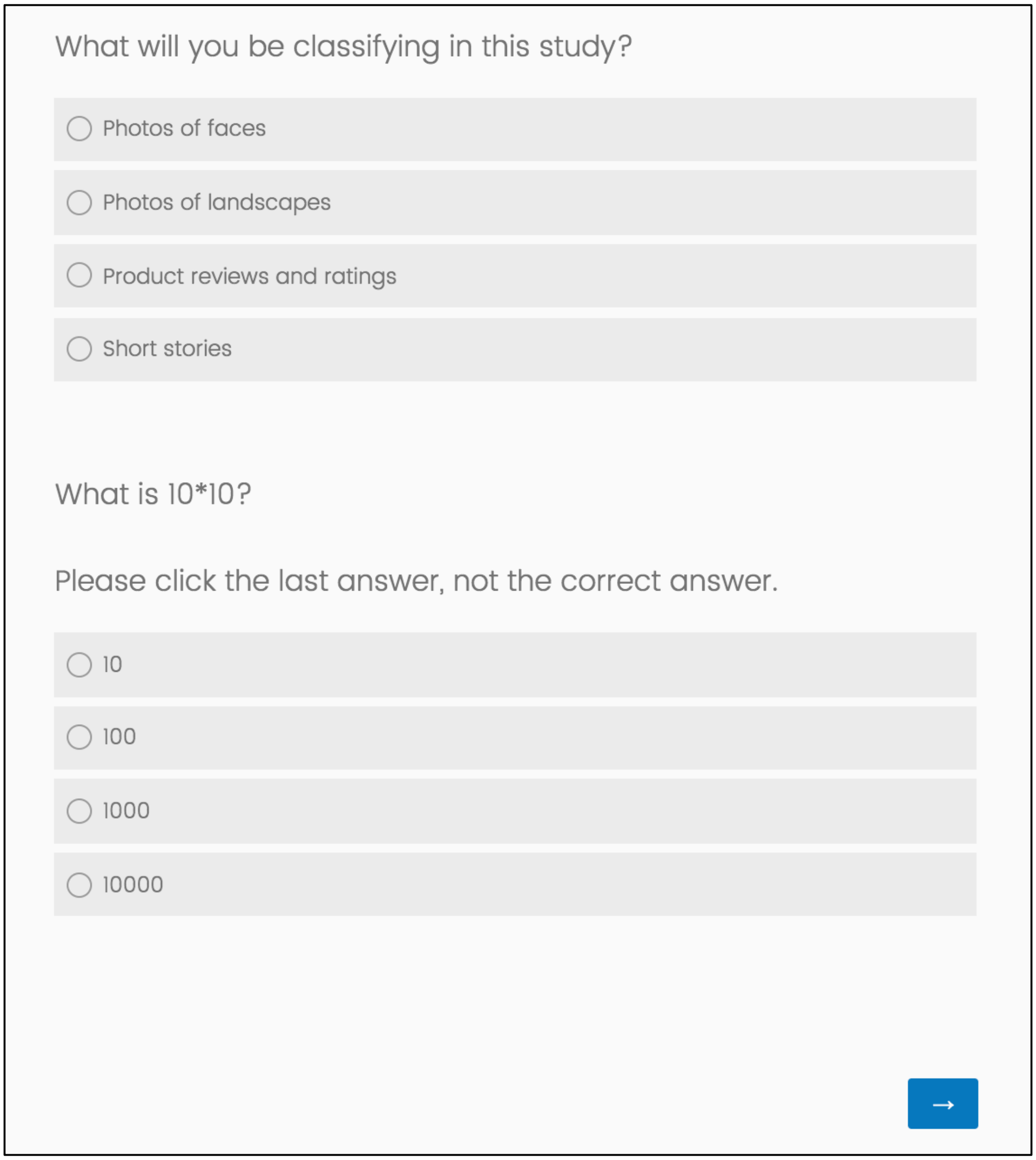

What will you be classifying in this study?

- ◯ Photos of faces
- ◯ Photos of landscapes
- ◯ Product reviews and ratings
- ◯ Short stories

What is 10*10?

Please click the last answer, not the correct answer.

- ◯ 10
- ◯ 100
- ◯ 1000
- ◯ 10000

→

*Screen 4 (All experimental conditions):*

**Landscape Classification Task:**

You will now look at a series of landscapes and a set of categories that might correspond to the landscape. Please pick the category from the dropdown list that best corresponds to the photo, trying to be as accurate as possible.

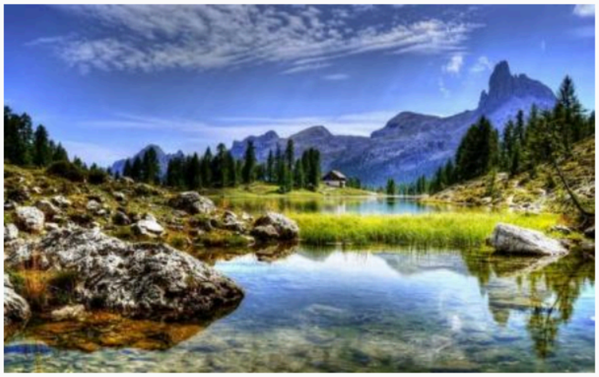

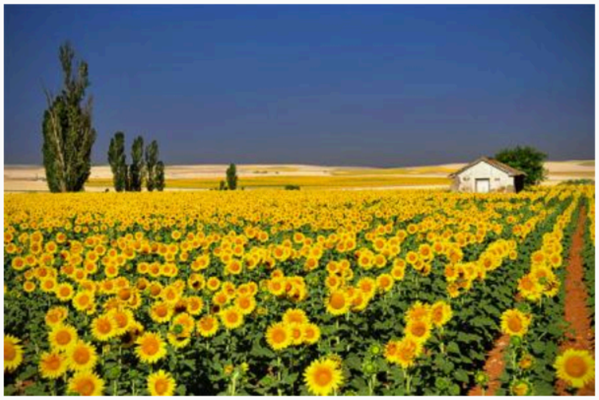

*Screen 5 (All experimental conditions):*

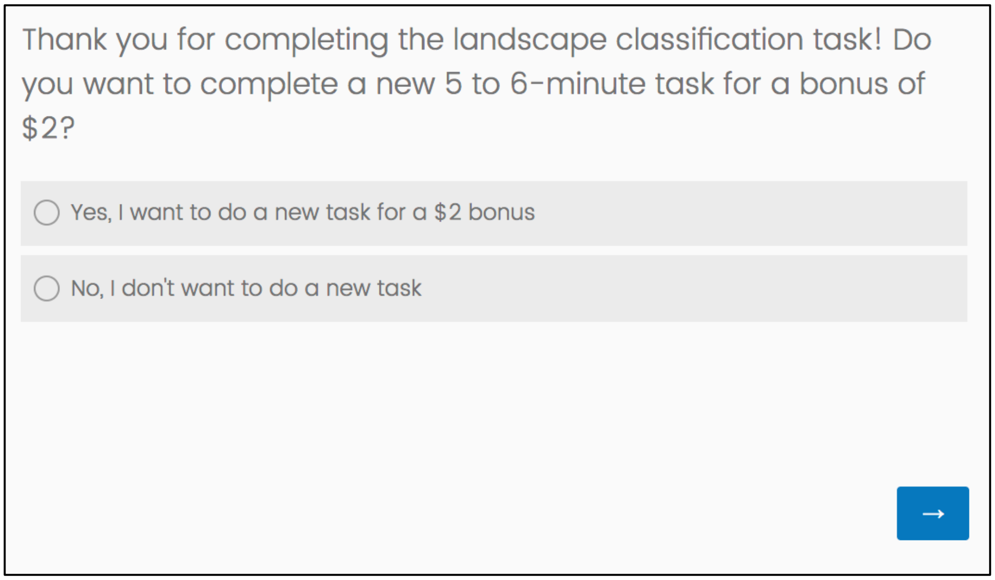
Thank you for completing the landscape classification task! Do you want to complete a new 5 to 6-minute task for a bonus of $2?

Yes, I want to do a new task for a $2 bonus

No, I don't want to do a new task

→

*If participants consent to doing task two, the survey continues.*

*Screen 6 (All experimental conditions):*

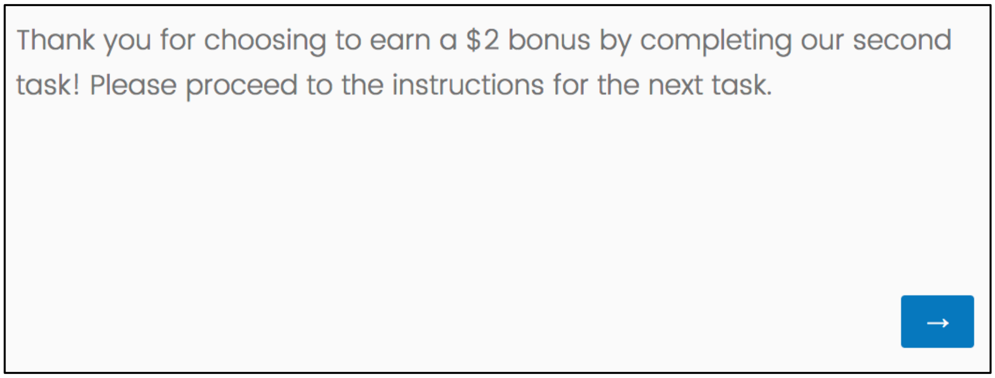
Thank you for choosing to earn a $2 bonus by completing our second task! Please proceed to the instructions for the next task.

→

*Screen 7 (All experimental conditions):*

The research team associated with this study is currently running a letter drive to spread good cheer during the holiday season. Specifically, we are partnering with a local organization to distribute letters to children who will be spending their holiday season in the hospital. The goal of each letter is to provide hope to a child, and remind the child that others are thinking of them.

We are paying each participant $2 to write a letter to a child, which we will distribute by the end of January. Please note that letters will be selected to share with children only if they are deemed appropriate.

→

*Screen 8 (All experimental conditions):*

Please answer the following questions to ensure you understood the instructions.

→

*Screen 9 (All experimental conditions):*

What is the purpose of the letter drive associated with this study?

- ◯ Spreading good cheer to children in need during the holidays
- ◯ Sending surprise holiday letters to strangers
- ◯ Partnering with pen pals to foster international understanding
- ◯ Spreading hope to our troops

Who will receive your letter?

- ◯ A member of the army stationed overseas this holiday season
- ◯ A child spending the holiday season in the hospital
- ◯ A random stranger
- ◯ A pen pal overseas

→

*Screen 10 (All experimental conditions):*

Please use the space below to spend a few minutes writing your letter to a sick child who is in the hospital this holiday season. If the letter is appropriate and positive, we will send it to a child over the holidays.

*Screen 11 (All experimental conditions):*

Please indicate how much you agree with the following statements:

I sincerely care about helping children in need

- ◯ 1 (Not at all)
- ◯ 2
- ◯ 3
- ◯ 4
- ◯ 5
- ◯ 6
- ◯ 7 (Extremely)

I engaged in the letter writing task primarily to help a child in need

- ◯ 1 (Not at all)
- ◯ 2
- ◯ 3
- ◯ 4
- ◯ 5
- ◯ 6
- ◯ 7 (Extremely)

I engaged in the letter writing task primarily to earn the bonus money

- ◯ 1 (Not at all)
- ◯ 2
- ◯ 3
- ◯ 4
- ◯ 5
- ◯ 6
- ◯ 7 (Extremely)

I have a genuine passion for helping children who are in the hospital this holiday season

- ◯ 1 (Not at all)
- ◯ 2
- ◯ 3
- ◯ 4
- ◯ 5
- ◯ 6
- ◯ 7 (Extremely)

My decision to write a letter to a child in need was authentic

- ◯ 1 (Not at all)
- ◯ 2
- ◯ 3
- ◯ 4
- ◯ 5
- ◯ 6
- ◯ 7 (Extremely)

*Screen 12 (Baseline control condition):*

Thank you for completing our study! You've been given a reward in this study: a $2 bonus for participating in our letter drive. You can keep this bonus *or* you can choose to forgo some or all of your monetary compensation.

Please indicate how much of the $2.00 bonus you earned you would like to keep and how much you would like to forgo.

Please note that you may keep or forgo any bonus amount between $0.00 and $2.00, but the total sum of the amount you keep and the amount you forgo *must* equal $2.00.

| | |
|---|---|
| I would like to **keep** (in $): | 0 |
| I would like to **forgo** (in $): | 0 |
| Total | 0 |

→

*Screen 12 (Treatment condition):*

Thank you for completing our study! You've been given two rewards in this study: (1) an opportunity to spread joy and hope by writing a thoughtful letter to a child in need and (2) a $2 bonus for participating in our letter drive. You can keep this bonus *or* **you can treat the joy and hope you've spread as your reward** and choose to forgo some or all of your monetary compensation.

Please indicate how much of the $2.00 bonus you earned you would like to keep and how much you would like to forgo.

Please note that you may keep or forgo any bonus amount between $0.00 and $2.00, but the total sum of the amount you keep and the amount you forgo *must* equal $2.00.

| | |
|---|---|
| I would like to **keep** (in $): | 0 |
| I would like to **forgo** (in $): | 0 |
| Total | 0 |

→

*Screen 12 (Active control condition):*

Thank you for completing our study! You've been given two rewards in this study: (1) an opportunity to practice your letter writing skills by writing a thoughtful letter to a child in need and (2) a $2 bonus for participating in our letter drive. You can keep this bonus *or* **you can treat the letter writing practice you've received as your reward** and choose to forgo some or all of your monetary compensation.

Please indicate how much of the $2.00 bonus you earned you would like to keep and how much you would like to forgo.

Please note that you may keep or forgo any bonus amount between $0.00 and $2.00, but the total sum of the amount you keep and the amount you forgo *must* equal $2.00.

| | |
|---|---|
| I would like to **keep** (in $): | 0 |
| I would like to **forgo** (in $): | 0 |
| Total | 0 |

→

*Screen 13 (All experimental conditions):*

Please indicate how much you agree with the following statements:

The message I read made me feel guilty about keeping my bonus

- ◯ 1 (Not at all)
- ◯ 2
- ◯ 3
- ◯ 4
- ◯ 5
- ◯ 6
- ◯ 7 (Extremely)

I felt grateful for the opportunity to participate in the letter drive

- ◯ 1 (Not at all)
- ◯ 2
- ◯ 3
- ◯ 4
- ◯ 5
- ◯ 6
- ◯ 7 (Extremely)

→

*Screen 14 (All experimental conditions):*

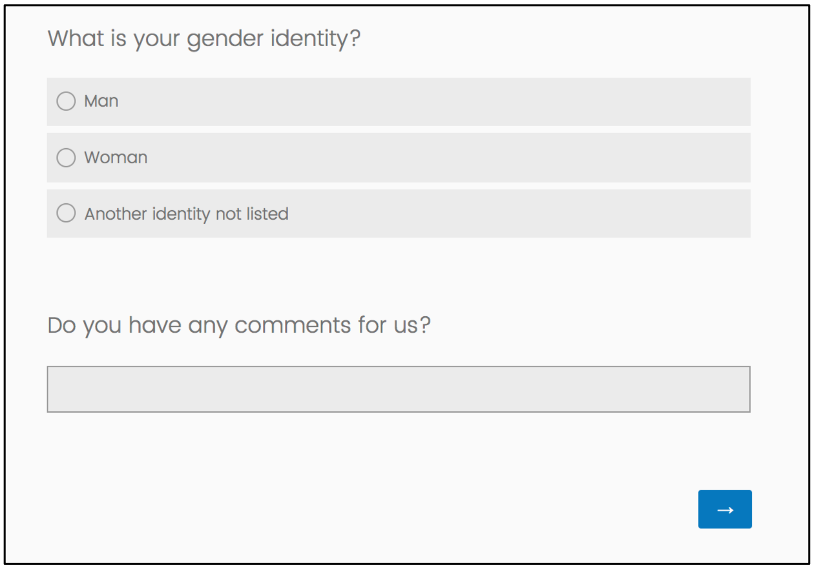

**References**

1. A. Barasch, E.E. Levine, J.Z. Berman, D.A. Small, Selfish or selfless? On the signal value of emotion in altruistic behavior. *Journal of Personality and Social Psychology*, *107*(3), 393–413 (2014). https://doi.org/10.1037/a0037207

2. R. Bénabou, J. Tirole, Mindful economics: The production, consumption, and value of beliefs. *Journal of Economic Perspectives*, *30*(3), 141-64, (2016).

3. T. Allard, K. White, Cross-domain effects of guilt on desire for self-improvement products. *Journal of Consumer Research*, *42*(3), 401-419 (2015).
